# Supplementary figures and images for: Pathways to scale up early childhood programs: A scoping review of Reach Up and Care for Child Development
Source: PLOS Glob Public Health. 2023 Aug 9;3(8):e0001542. doi: 10.1371/journal.pgph.0001542 (PMC10411826; doi:10.1371/journal.pgph.0001542)

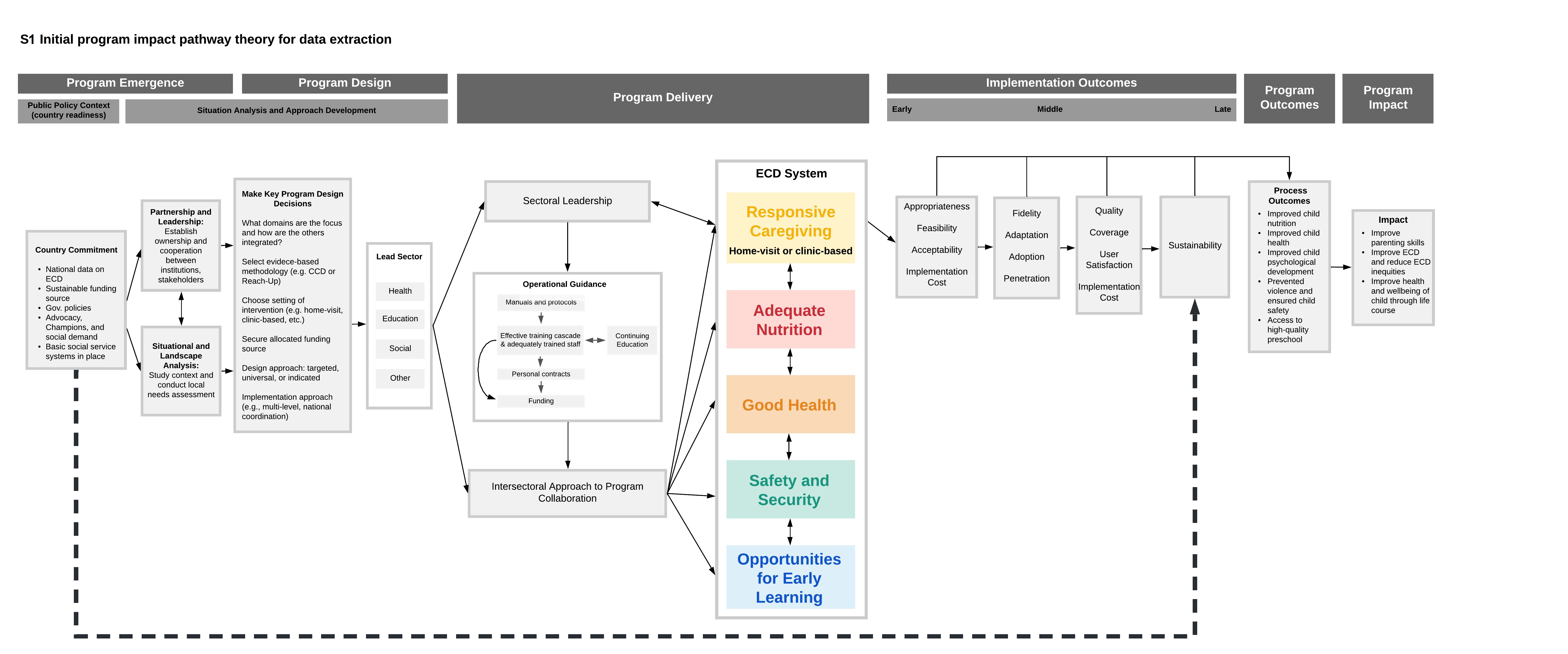

Supplement: S1 Fig — (TIFF) [file pgph.0001542.s002.tiff]
