# Supplementary material for: Pathways to scale up early childhood programs: A scoping review of Reach Up and Care for Child Development
Source: PLOS Glob Public Health. 2023 Aug 9;3(8):e0001542. doi: 10.1371/journal.pgph.0001542 (PMC10411826; doi:10.1371/journal.pgph.0001542)
Supplement: S1 Table — (DOCX) [file pgph.0001542.s003.docx]

| **S1 Table. Description of RU and CCD Methodologies using the Template for Intervention Description and Replication (TIDieR).** | | |
| --- | --- | --- |
| Name | Reach Up and Learn (RU) | Care for Child Development (CCD) |
| Why | Interventionists build positive relationships with caregivers and aim to strengthen caregiver skills, self-esteem, and enjoyment in helping their child. Trained home visitors’ partner with caregivers by consulting on their experience with caregiving, asking their opinions, and listening to concerns and priorities. RU uses an interactive approach of modeling and practicing activities as a form of skill-building. Home visitors emphasize praise for caregivers and children. | Program facilitators are equipped to identify relevant interactions between a child and their caregiver, counsel families on activities that could help strengthen their relationship with their children, and advise families on how to best promote their child's optimal growth and healthy development through activities. |
| What | Materials: Manuals for home visits, toy construction, adaptation and planning guide, training with accompanying films, and supervision. Locally made play materials include toys, pictures, puzzles, and classification games.  Procedures:  1. Time to check in with family and their progress.  2. Home visitor introduces new activities in an interactive way.  3. Home visitor observes the child's behaviors and developmental skills.  4. Demonstration and description of activity to caregiver and child; help child with activity.  5. Encourage caregiver and child to practice; give positive feedback and celebrate success.  6. Review of activities for caregivers to continue during week and encourage caregivers. | Materials: Homemade toys and household objects; manuals that summarize course content, discussions and exercises; counselling cards recommending activities for facilitators to teach caregivers to foster motor, social, cognitive, and emotional development in children; checklist to evaluate how child is being cared for during home visits and to inform if/how to improve relationships between the children and their caregivers.   Procedures:  1. Providers greet caregivers and ask about how they typically interact with their child.  2. Provider observes how the caregiver responds to, comforts, demonstrates love, and guides the child's growth.  3. Provider uses information from the caregiver and age-appropriate information on development to praise the caregiver, build the caregiver's confidence, increase how the caregiver talks to the child, and identify activities for the caregiving to do with the child.  4. Problem solving issues the caregiver brings up.  5. Schedule follow-up visits to encourage the caregiver to engage in new caregiving tasks identified during the visit. |
| Who | Home Visitors: Implementation agents with a minimum of completed primary education; can be newly-trained RU implementation agents or RU can be integrated into the workload of existing staff at health centers, ECD centers, or other organizations.  Training: 10-day interactive training with in-person interaction, films, role-playing, and small-group activities. Training covers basics of ECD, importance of caregiving, how to conduct a successful home visit using the provided curriculum, how to demonstrate activities, and toy construction. At the end of training home visitors are accompanied on a practice home visit. | Home Visitors: Providers can be trained from a range of sectors including health, education, nutrition, childcare, emergency services, child protection, and other family services to conduct CCD sessions that can occur in homes or other settings. Providers may also be trained in WHO Integrated Management of Childhood Illness (IMCI).  Training: Three-day training implemented by two facilitators for groups of twelve participants and guided by a training manual, facilitator notes, CCD manual, PowerPoint presentations. |
| How | Individual home visits with implementation agents, child's caregiver, and child. | In-person visits between the provider and caregiver. |
| Where | Low- and middle-income countries. Lead implementing organization depends on the context. Program can be integrated with other services (e.g. nutrition) depending on local priorities. | Low- and middle-income countries.  Intervention can be delivered in home, clinical, or other public space settings. The health system is identified as a common platform in place in low- and middle-income countries with the potential to integrate responsive caregiving activities. |
| When and How Much | Weekly, 1-hour home visits from child's birth until 3 years of age. | The delivery setting determines the frequency and duration of visits. |
| Tailoring | Program should integrate other national priorities in addition to using locally available materials to make cost-effective play materials, and including culturally appropriate images and local songs. | Program can be integrated into existing programs such as home visits, parent groups, childcare centers, or nutrition programs. Training and visit contents can be adapted to local conditions. |
| How Well | Supervisors provide ongoing support and monitoring of implementation agents, should accompany implementation agents once per month on home visits, and use a checklist to monitor home visit quality and fidelity. | There is a CCD monitoring and evaluation framework the outlines how to monitor implementation status and quality as well as evaluate impact |
| ^a^ “Modifying” item of TIDieR was not applicable to this description | | |
