# Supplementary material for: Pathways to scale up early childhood programs: A scoping review of Reach Up and Care for Child Development
Source: PLOS Glob Public Health. 2023 Aug 9;3(8):e0001542. doi: 10.1371/journal.pgph.0001542 (PMC10411826; doi:10.1371/journal.pgph.0001542)
Supplement: S2 Table — (PDF) [file pgph.0001542.s004.pdf]

S2 Table. Search Terms

Population: RU or CCD	Context: Implementation	Concept: Multisectorality
("Reach up"	AND ("implementation facilitat*"	AND ("nurturing care"
OR "Care for Child Development"	OR "implementation barrier*" OR "barriers to implemen*"	OR "nurturing interaction*"
OR "Care for Development"	OR "implementation science*"	OR multisectoral*
OR "Chile Crece Contigo"	OR "implementation strateg*" OR "implemented strateg*"	OR intersectoral*
OR "Jamaican Intervention"	OR "implementation outcome*"	OR "multiple sector*"
OR "Caring for the Child's Healthy Growth and Development"	OR "implementation challenge*"	OR "responsive caregiving"
OR "China REACH"	OR "implementation evaluation"	OR "integrated intervention*"
OR "Rural Education and Child Health Project"	OR "intervention adopt*" OR "adopted intervent*"	OR "integrated approach*"
OR "Cuna Mas"	OR "implementation research*"	OR "integrated system*"
OR "Learning Club*"	OR spread	OR "integrated service*"
OR Urban95	OR sustain*	OR "integrated strateg*"
OR Istanbul95	OR uptake	OR "integrated program*"
OR "Sugira Muryango"	OR penetration	OR "integrated curricul*"
OR Anganwadi	OR acceptability	OR "different delivery strateg*"
OR "Family Women and Infancy Program"	OR acceptable	OR "multicomponent intervention")
OR "early child development"	OR appropriate*	
OR "early childhood"	OR feasib*	
OR "home visit*"	OR fidelity	
OR "home based intervention"	OR efficien*	
OR "parenting skill*"	OR "patient-centered"	
OR "psychosocial intervention"	OR adapt*	
OR "responsive caregiv*"	OR dissemin*	
OR "responsive parent*")	OR "enabling environment"	
	OR efficac*	
	OR "contextual factor*"	
	OR "process evaluation")	
