# Supplementary material for: Pathways to scale up early childhood programs: A scoping review of Reach Up and Care for Child Development
Source: PLOS Glob Public Health. 2023 Aug 9;3(8):e0001542. doi: 10.1371/journal.pgph.0001542 (PMC10411826; doi:10.1371/journal.pgph.0001542)
Supplement: S3 Table — (DOCX) [file pgph.0001542.s005.docx]

**S3.** Data Extraction Items

| **Domain** | **Component** | **Definition** |
| --- | --- | --- |
| Context | World region | Region classified using WHO world regions [(1)](https://www.zotero.org/google-docs/?4DzgOm). |
|  | Country income level | Income level classified using World Bank country and lending groups [(2)](https://www.zotero.org/google-docs/?gEr7Gp). |
|  | Years of implementation | Year of program initiation and end. |
|  | Program aims | Implementation goal and outcome goal of the program. |
|  | Implementation scale | One scalable unit is a one administrative district with all infrastructural components critical to the intervention where the intervention can be fully delivered. This component captures the scale of implementation by assigning a category: single scalable unit, multiple scalable units, full scale. Full scale indicates the intervention is delivered to a large number of sites with different contexts [(3)](https://www.zotero.org/google-docs/?WtUg36). |
|  | Reach | Reach is the number of unique individuals affected by a program. In this case, we use the number of children receiving the intervention because there typically was not enough data to estimate the number of caregivers [(4)](https://www.zotero.org/google-docs/?aDHoEf). |
|  | Child age | Age of children who are enrolled in and receive the program. |
|  | Site Urbanization | Urbanization level of area in which intervention is being implemented: rural areas, towns and semi-dense areas, or cities [(5)](https://www.zotero.org/google-docs/?HRt41k). |
|  | Length | Course of time over which the intervention was delivered. |
|  | Country situation | Country-level context such as background data or policy context that are relevant to ECD. |
| Implementation Strategies | ECD systems | Existing government social services, policies, and funding that directly supported the program emergence of RU implementation. |
|  | Sectoral leadership and partnership | Which sectors take the lead of ECD programs and who they partner with such as the health sector, education sector, social assistance sector, or other institutions such as universities or non-governmental organizations. |
|  | Funding | Sources of funding for program implementation. |
|  | Focal nurturing care domains | Program focuses on one or multiple nurturing care domains. |
|  | Intersectoral approach to program collaboration | How multiple sectors collaborate for intervention delivery and how the program delivers intervention components related to multiple nurturing care domains. |
|  | Intervention setting | The setting of intervention delivery can include participant homes or local centers; intervention can be delivered to individual caregiver-child pairs or in group settings. |
|  | Dose | The amount of intervention delivered operationalized as time spent for each home visit or parenting skill session [(6)](https://www.zotero.org/google-docs/?K2ev2r). |
|  | Frequency | How often visits are delivered to program beneficiaries. |
|  | Design approach | Targeting protocol of the program is classified into one of the following:  (1) universal: program delivered to the entire population  (2) selective: program delivered to sub-populations  (3) indicated: program delivered to individuals who have been screened and determined to meet specific eligibility criteria [(7)](https://www.zotero.org/google-docs/?zmwSjr). |
|  | Materials | Toys, books, puzzles, and other materials and how they were used during program sessions. |
|  | Number of implementation agents | How many people are trained and deliver the program at a given time. Implementation agent refers to the people working as home visitors or who deliver CCD or RU in a public or group setting outside of family homes [(8)](https://www.zotero.org/google-docs/?l0y2oP). |
|  | Implementation agent characteristics and recruitment | The traits of the trained implementers, home visitor recruitment strategy, and eligibility criteria. |
|  | Implementation agent caseload | The number of families for which each implementation agent is responsible. |
|  | Personal contracts and compensation | The financial compensation structure for implementation agents. |
|  | Training manuals and protocols | The instructional materials staff use in training and ongoing operations. |
|  | Training | Length of implementation agent training. |
|  | Supervision and mentorship | Characteristics of intervention agent supervisors and the characteristics of supervisory interactions with implementation agents. |
|  | Supervisor characteristics and recruitment | The traits of the trained supervisors, recruitment strategy, and eligibility criteria. |
|  | Supervisor training | The length, content, and training strategies of supervisors. |
|  | Number of supervisors | The total number of trained and active supervisors supporting the program. |
|  | Supervisor caseload | The number of implementation agents for which each supervisor is responsible. |
|  | Workforce mentorship | How supervisors monitor, support, and coach implementation agents on program delivery activities. |
|  | Program data and reporting | The process by which program data were collected, organized, maintained, and reported. |
| Implementation Outcomes | Appropriateness | Perceived fit, relevance, or compatibility of the innovation for a given setting or population [(9)](https://www.zotero.org/google-docs/?NslpGo). |
|  | Feasibility | The extent to which a new innovation can be successfully implemented in a given setting [(9)](https://www.zotero.org/google-docs/?lmQKKN). |
|  | Acceptability | A given service is agreeable, palatable, or satisfactory [(9)](https://www.zotero.org/google-docs/?Pp9FrI). |
|  | Adoption | Intention, initial decision, or action to employ an innovation or practice, i.e. the uptake [(9)](https://www.zotero.org/google-docs/?5GMHIK). |
|  | Fidelity | The degree to which an intervention was implemented as it was prescribed and intended in the original protocol [(9)](https://www.zotero.org/google-docs/?NzhzOm). |
|  | Adaptation | Changes made to an intervention are based on deliberate considerations to increase fit with a patient or contextual factor [(10)](https://www.zotero.org/google-docs/?BcZQWh). |
|  | Penetration | Integration of practice within a service setting [(9)](https://www.zotero.org/google-docs/?qZgfmw). |
|  | Sustainability | The extent to which a newly implemented treatment is maintained or institutionalized within a service setting’s ongoing operations [(9)](https://www.zotero.org/google-docs/?3K7tiO). |
|  | Implementation cost | The cost impact of an implementation effort [(9)](https://www.zotero.org/google-docs/?vVXaSf). |
|  | Scaling | Deliberate efforts to increase the impact of health service innovations to benefit more people and foster policy and program development on a lasting basis [(3)](https://www.zotero.org/google-docs/?HDzBup). |
|  | Program outcomes | Observed effects of the program on domains of early childhood development. |
|  | Impact | Long-term results of a program on early childhood development. |

References:

[1. Countries | World Health Organization [Internet]. [cited 2021 Jun 17]. Available from: https://www.who.int/countries](https://www.zotero.org/google-docs/?CwlaPi)

[2. World Bank Country and Lending Groups – World Bank Data Help Desk [Internet]. [cited 2021 Jun 17]. Available from: https://datahelpdesk.worldbank.org/knowledgebase/articles/906519-world-bank-country-and-lending-groups](https://www.zotero.org/google-docs/?CwlaPi)

[3. Barker PM, Reid A, Schall MW. A framework for scaling up health interventions: lessons from large-scale improvement initiatives in Africa. Implementation Science. 2016 Jan 29;11(1):12.](https://www.zotero.org/google-docs/?CwlaPi)

[4. Centers for Disease Control and Prevention. CTG Program Reach Guidance. US Department of Health and Human Services; 2012.](https://www.zotero.org/google-docs/?CwlaPi)

[5. European Commission. A recommendation on the method to delineate cities, urban and rural areas for international statistical comparisons [Internet]. 2020. Available from: https://ec.europa.eu/eurostat/cros/system/files/bg-item3j-recommendation-e.pdf](https://www.zotero.org/google-docs/?CwlaPi)

[6. Rowbotham S, Conte K, Hawe P. Variation in the operationalisation of dose in implementation of health promotion interventions: insights and recommendations from a scoping review. Implementation Science. 2019 Jun 6;14(1):56.](https://www.zotero.org/google-docs/?CwlaPi)

[7. Gordon RS. An operational classification of disease prevention. Public Health Rep. 1983 Apr;98(2):107–9.](https://www.zotero.org/google-docs/?CwlaPi)

[8. Dunst CJ, Trivette CM, Raab M. An Implementation Science Framework for Conceptualizing and Operationalizing Fidelity in Early Childhood Intervention Studies. Journal of Early Intervention. 2013 Jun 1;35(2):85–101.](https://www.zotero.org/google-docs/?CwlaPi)

[9. Proctor E, Silmere H, Raghavan R, Hovmand P, Aarons G, Bunger A, et al. Outcomes for implementation research: conceptual distinctions, measurement challenges, and research agenda. Adm Policy Ment Health. 2011 Mar;38(2):65–76.](https://www.zotero.org/google-docs/?CwlaPi)

[10. von Thiele Schwarz U, Aarons GA, Hasson H. The Value Equation: Three complementary propositions for reconciling fidelity and adaptation in evidence-based practice implementation. BMC Health Services Research. 2019 Nov 21;19(1):868.](https://www.zotero.org/google-docs/?CwlaPi)
