# Supplementary material for: Pathways to scale up early childhood programs: A scoping review of Reach Up and Care for Child Development
Source: PLOS Glob Public Health. 2023 Aug 9;3(8):e0001542. doi: 10.1371/journal.pgph.0001542 (PMC10411826; doi:10.1371/journal.pgph.0001542)
Supplement: S6 Table — (PDF) [file pgph.0001542.s008.pdf]

S5a Table. Implementation Outcomes of Care for Child Development Programs

Country	Program Name	Appropriateness	Feasibility	Acceptability	Adoption	Fidelity	Adaptation	Penetration	Sustainability	Implementation Cost	Scaling	Program Outcomes
**African Region**												
**Kenya** (low income)	Msingi Bora	Caregivers: The intervention included a father involvement arm in which fathers were actively encouraged to participate and the program included father only sessions covering topics such as respectful communication, father engagement in caregiving, and emotional support between partners. Comprable sessions were delivered to mothers at the same time. Despite this stragety, father attendance was low attending a median of just one of the sixteen sessions. 52% of fathers attended at least one session. Future work should focus on engaging fathers more effectively in the sessions and caregiving.	Caregivers/Workforce: Group based intervention is as effective or more effective than mixed intervention with individual home visits demonstrating a less burdensome, more feasible way to deliver ECD services. One theory for why group sessions had greater impact on child development is the role of peer learning during group review sessions.	Caregivers: Mothers reported satisfaction with the group sessions particularly pointing to their appreciation of interacting with others in the groups, learning from each others' questions and answers and the interactions of other mothers with their children.	Not reported	Workforce: SWAP staff served as supervisors and monitored community health volunteers' key skills and overall session quality. The monitoring data confirmed the sessions were delivered as intended. 89% of group sessions were monitored and 9% of home visits were monitored. Community health volunteers also took attendance sheets to confirm session delivery. Supervisors provide feedback to home visitors following sessions. This feedback included comments on skills of facilitating discussion, coaching parents, andwering questions, and overall session quality.	Adapted the program to be more appropriate to fathers but this was not fully successful. Four sessions were designed to be attended exclusively by male caregivers covering issues such as father engagement and barriers, respectful communication with partners, emotional regulation and support.	Not reported	Sustainability of impact depends of caregivers incorporating activities into their daily behaviors. This could be encouraged by periodic reviews of ECD material and activities during booster sessions.	The use of group sessions as a delivery platform is more cost effective than individual home visits. A time use survey showed that individual home visits used an additional 27 hours compared to one group session. Group sessions massively save opportunity cost of delivery agents' time.	The group sizes had a median of 13 mothers which is relatively large and demonstrates a way to feasibly scale ECD programs being efficient with resources. Additionally, this demonstrated the use of existing community health volunteers and NGO infrastructure to be incorporated into novel ECD programs.	Children in the group only treatment arm had improvements in cognitive, language, and socioemotional development. Children in the mixed-delivery arm improved in these areas as well compared to the control, but did not improve as much as the group arm. Higher levels of compliance and attendance resulted in greater developmental gains. Children in treatment arms had improved HOME scores. Children in group arm had improved dietary diversity. No improvement in child stunting in either group. There were imprvements in maternal knowledge of development but no improvement in maternal wellbeing.
	Smart Start Siaya County (PATH)	Community health workers, other public workers, and families were all accepting of Smart Start and shifted their understandings of ECD.	Program emergence: Joint planning among partners was cited as crucial to successful program implementation.	Workforce: Community health volunteers accepted their position with the formal compensation relieving ongoing economic pressure and giving them more time to do nurturing care activities. There are several challenges that limit the acceptance of community health workers of their role: minimal recognition (no salary before 2015) and significant responsibilities, lack of training and resources, limited incentives. The introduction of community health worker stipends improved acceptability. This improved job satisfaction and performance. Nurses implementing nurturing care activities were more accepting toward adding new activities into their workflow after seeing the positive benefits and viewing new activities as shifting the way they work, not adding new work.<br><br>A growing number of men in the community were accepting of engagement with the program and father roles in caregiving thanks to the role of male community leaders. Community health workers, other public workers, and families were all accepting of Smart Start and shifted their understandings of ECD.	A multi-sectoral team was established made up of officials from each ministry within the county government. The team convenes for one day every four months and uses data and information compiled by the technical working group. Partner organizations include NGOs such as PATH and Unicef. On a governmental level, ECD is considered the responsibility of the ministry of education, but the ministry of health has filled in gaps of the MoE not prioritizing pre school aged children.	Not reported	Culturally-specific training on evidence-based techniques could help community health workers improve their work with caregivers. PATH took the lead adapting CCD to this context.	75% of health facilities have a health provider trained on CCD. 25% of county's 2,148 community health workers are trained on CCD. Every sub-county has a provider implementing CCD.	The payment of the community health worker was critical to strong reporting.<br><br>Programming has been led by the County Governor and First Lady of Siaya County, who earmarked nurturing care as an essential project to be prioritized to achieve other development goals in Siaya.<br><br>Community health assistants who are certified nursing or public health workers are based at health clinics and supervise the community health workers. New supervisors called mentor coordinators were hired to supervise and complement the existing supervisors. Mentor coordinators identified training needs, collecting data and monitor quality, and coaching.	CCD training was integrated into existing workforce and training, maximizing cost efficiency.<br><br>Despite the national policy, not all counties possess the technical and financial resources to enact policy frameworks into practice.	Local government and community ownership of Smart Start is critical for scaling within the geographic area. The strong leadership from the county government has enabled the success of the program at the county scale.<br><br>Scaling was also enabled by the use of an existing health system platform.<br><br>Three more potentials for scaling up include: providing more in-depth training to staff on nurturing care (e.g. family conflict resolution), integrating nurturing care into a more diverse spread of government activities, and replicating smart start in other counties in Kenya. The leadership of the county demonstrates innovation that can be transfered to other counties. Neighboring counties including Turkana County are [illegible]	Not reported
**Malawi** (low income)	CCD	Program design: There was a higher attendance rate in the urban setting than the rural setting indicating more barriers in rural settings. Barriers included other commitments such as funerals and the need to work in agriculture. The program was implemented during harvest season which exacerbated this barrier. (program emergence)<br><br>Caregivers: Healthcare workers identified that the core content of the CCD package was repetitive to parents when provided twice a month and that the content needed to be developed or changed further over time.)	Home visit duration was decreased due to long travel times and distances in rural settings. It was not feasible to use HSAs to deliver CCD due to their existing heavy workload of providing other health services. Feasibility could improve if a selective design approach was used to enroll only the most vulnerable children and providing a smaller caseload for each home visitor.	Caregivers: The program was specifically adapted based on extensive formative research including interviews and focus groups with 180 caregivers and healthworkers making the content acceptable to caregivers. Parents and caregivers knew the health surveillance assistants and the role they played in the community, making them familiar and approachable entities. Some female caregivers indicated that a barrier to their participation was receiving services from the majority male health surveillance assistants. It was not culturally appropriate for a woman at home to receive visits from a non-relative male on a one to one basis. Men were perceived to be good at leading things, but some mothers thought the service should be provided by women as it was a topic that dealt with women. Caregivers indicated satisfaction with mixed delivery as group sessions provided opportunities for shared learning and community cohesion and individual sessions provide privacy. Sometimes group sessions led mothers to be embarassed if, for example, they came to the session and their child was hungry. Participants (caregivers) felt the community held negative views of the intervention.<br>Workforce: Some health surveillance assistants indicated that they felt they were not fully equiped and that the additional workload was too much on top of their existing tasks.	Of the families contacted to enroll in the program, only one family refused. Only 41% of urban caregivers and 16% of rural caregivers attended over 75% of the group sessions. In both sites, about two thirds of caregivers attended over 50% of the planned individual sessions. Health surveillance assistants carried out 87% of the group sessions and 67% of individual sessions.	Fidelity was measured by evaluating data collection forms filled out by the health surveillance assistants after sessions that included information on the atendees, cancellation of sessions, and barriers to attendance. Research assistants also observed two group and two individual sessions for each health surveillance assistant to measure quality and fidelity. These methods revealed that health surveillance assistants were mostly good at encouraging caregivers, only brought example play materials less than half of the time, and sometimes appeared bored.	Adapting CCD to take into account the social milieu and specific cultural and social barriers to care of young children in Malawi made the program more acceptable to caregivers. Specifically, the package was adapted to include nutrition and water, sanitation, and hygiene advice because that was indicated as an interest.	Using health surveillance assistants it was only feasible to provide services to 14.2% of children under the age of two within the catchment areas indicating a low reach with this delivery mechanism.	Not reported	Not reported	For workforce: Most importantly, a different cadre of workers would have to be used, such as women's groups, as the health surveillance assistants were overloaded. health surveillance assistants could serve a supervisory role to women's groups.<br>For families: The intervention would have to be delivered at times and locations more convenient to mothers.	Children receiving CCD had significant improvement in language and social development indicators and home stimulation.
**Mozambique** (low income)	Nurturing Care Collaboration (PATH)	Caregivers: some messages could not be understood by caregivers, some household were too far from health facilities and limited presence of community providers; lack of male caregiver engagement in children's health services.	Program integration was found to be feasible in low-resource settings.	Workforce: The adapted materials have been well received by the CHWs and clinicians. Providers were satisfied with training and supervision received. Program content was considered useful.<br>Caregivers: Program was found to improve caregivers' perceptions of service quality. Perception of usefulness of messages on nutrition and parenting. Reported satisfaction with couseling received from health facility providers. Dissatisfaction with long waiting times at health facilities.	Strong leadership and support from goverment district to enhance nurturing care as essential to the overall health system. Inadequate resources to carry out responsabilities; increased responsabilities; low motivation among providers; language barriers; weak linkage between facilities and community providers. Training and supervision to providers as well as provision of materials and job aids facilitated adoption.	PATH and government mentors use a 24 question checklist to monitor fidelity to CCD and other program aims.	The CCD participant manual and counselling cards were adapted to meet the unique needs of families in Mozambique. Specifically, due to the high level of illiteracy and linguistic diversity the materials were adapted to be primarily visual using pictures.	The health system was used as an entry point for promoting and integrating ECD services throughout the health system, supporting the role of the health sector in integrating ECD programs in low-resource settings, and strengthening the enabling environment through inclusion of ECD policy, guidelines, training curricula, and governmental plans and budgets.	Ownership by local government, community, and organization leaders is critical to sustained activities and effectiveness. Additionally, using existing health workforce is a way to facilitate sustainable implementation. Advocacy efforts resulted in ECD content inclusion in the pre-service training curricula of nurses and community health workers, helping reduce in-service training costs.	Not reported	Using existing health workforce was a way to facilitate rapid scaling. Three ways to continue to scale the program are: expanding community ownership and generating demand for ECD support potentially by collaborating with community and religious leaders, enhancing coordination for even coverage throughout the district (right now limited by rural geography), building government capacity to expand nurturing care initiatives to other sectors, and involving local businesses. The model of training CHWs and clinical providers in CCD is being expanded within Mozambique and in Kenya and South Africa.	Perceived change in efficience of child-centered services. Improved referral processes. Increased awareness of the health system's role in promoting ECD. Collection and monitoring ECD indicator in the health information system.
**Rwanda** (low income)	Sugira Muryango	Caregivers: A small number of caregivers dropped out due to time constraints. 80% of caregivers reported the intervention met their needs. The primary reason for not meeting needs being poverty; however, the program was generally appropriate for families living in poverty and may not be appropriate for other populations.<br>Workforce: The coach recruitment and compensation was designed to be comparable to existing volunteer workforce practices and be scalable. Conversations with caregivers and program staff revealed potential improvements including stronger linkages to local social supports for families, additional training and supervision for the coaches, a greater emphasis on father engagement, and integrating follow-up sessions to promote sustained impact.	The initial curriculum was 22 modules but was edited down to 15 for feasibility concerns and to ensure affordability. One challenge was actually linking caregivers to referral services in rural areas. While the pilot study used bachelors level interventionists, this trial trained community based coaches who effectively implemented the program, indicating that the intervention can be delivered by trained community members who do not need high levels of education. Caregivers in this intervention trial were equally satisfied as the caregivers who received the intervention from the bachelor-level coaches. This also demonstrated the feasibility to integrate the intervention into existing social protection programs. This allows the targetting of vulnerable families and addressing poverty as a root cause of ECD disparities.	Caregivers: 97% of caregivers are satisfied with the intervention including the information gained. Caregivers were equally satisfied with the bachelors level coaches as the community coaches.	Not reported	Fidelity was monitored by supervisor review of audio taped sessions. They promoted intervention fidelity and quality by providing tailored guidance via telephone to the coaches after listening to the tapes.	Community advisory boards and Rwandan government stakeholders provided input to adapt CCD to local contexts by using Rwandan songs and proverbs. The initial curriculum was 22 modules but was adapted and edited down to 15 for feasibility concerns and to ensure affordability.	Not reported	During the pilot trial, supervisors provided weekly phone supervision with implementation agents that consisted of reporting on activities, discussions as groups, and problem solving. A Rwandan program manager met with the "coaches" to review intervention delivery.<br><br>During the full trial, supervisors provided in-person supervision for three weeks after training and shadowed each CBC on one home visit. After that, they provided telephone supervision with peer support groups weekly. On a monthly basis, the supervisors facilitated a group discussion at the cell or sector office to address issues and problem solve as a group.<br>During the third and sixth week of the intervention, supervisors shadowed the implementation agents and provided immediate feedback.	Not reported	The coach recruitment and compensation was designed to be comprable to existing volunteer workforce practices and be scalable. Futher opportunity to scale-up the program could integrate resource navigation, booster sessions, and additional training and support. Future changes to improve scaling include making links to ECD centers, using both group and individual family sessions.	Intervention children had improved engagement in ECD activities at home, higher HOME scores, and greater OMCI scores. Intervention children has lower violence punishment but this was not sustained. There was not significantly different health or development outcomes. There was increased shared decision making and family unity among intervention families. There was lower serious family conflict in the intervention arm.
**Tanzania** (low income)	Integrated health, nutrition, responsive	Caregivers: After 18 months of implementation, 71% of women had received >90% of intended number of home visits.	Not reported	Caregivers: Some subgroups of women appeared to benefit more from the intervention, including who were married or cohabitating, and those with less than secondary education.	Not reported	Fidelity was monitored by supervisor review of notes taken by the community health worker. High fidelity in the frequency and length of home visits were found. Most women recalled receving advise on health, nutrition, and	The Tanzanian and Swahili adapted version of the UNICEF and WHO Care for Development package was used.	Not reported	Not reported	Not reported	Not reported	Mothers receiving the integrated health, nutrition, responsive stimulation packages reduced maternal depressive symptoms.
**Americas Region**												
**Brazil** (middle income)	Criança Feliz	Not reported	Operationalization of intersectoral collaboration has been identified as a major barrier during PCF design and implementation.<br><br>The rush to simultaneously scale up PCF and develop the training led to barriers in operating the multilevel training cascade capacity, resulting in an insufficient number of master trainers across the country and short-term consultants employed to be master trainers and developing training manuals. If these barriers were not addressed, they could negatively affect multilevel training cascade capacity and quality, and ultimately PCF effectiveness.	Caregivers: Families valued and perceived benefits from PCF. Some KIs reported an initial resistance from some families to engage with PCF due to poor communication of program goals that led families to wonder if the visits were a new requirement to continue receiving the conditional cash transfers from the Bolsa Familia and feel uncomfortable about having a home visitor every week engaging with family routines. However, these barriers were easily overcome after the initial home visits when families understood the goals of PCF and appreciated what it meant for the development of their children. Although difficulties in operationalizing, coordinating, and providing equitable, nurturing, family-centered care remain, according to KIs, the benefits of PCF perceived by the families have facilitated the scaling up (PFBL).	Federal intrasectoral challenges: Several federal intrasectoral barriers related to implementation capacity at early stages of PCF implementation generated an unfavorable institutional context for adoption within the existing SUAS<br><br>Intrasectoral resistance by states and municipalities: Some major barriers for adoption were weak social communication about ECD, political party opposition (e.g., a municipality led by a different political party than the federal government would not implement the program), lack of understanding and identification of the social assistance sector with PCF, as well as the delay of the federal-level leadership in reaching consensus on the messaging to communicate about PCF<br><br>WhatsApp communications and networks was a powerful enabler to overcome the initial resistance for PCF adoption by facilitating, enhancing, and accelerating communications and technical support.	Program emergence: Rushed scale: 3-month "strategic pause" to slow down the scale-up until minimum quality standards for operations were available. The absence of a user-friendly monitoring system for the program, in addition to heavy paperwork and the lack of implementation process indicators that stakeholders could refer to, emphasized the need for high quality training in order to make sure that fidelity implementation could be achieved in the different municipalities. The rush to simultaneously scale up PCF and develop the training led to barriers in operating the multilevel training cascade capacity, resulting in an insufficient number of master trainers across the country and short-term consultants employed to be master trainers and developing training manuals. If these barriers were not addressed, they could negatively affect multilevel training cascade capacity and quality, and ultimately PCF effectiveness. However, KIs perceived high-quality training as critical for achieving fidelity of implementation across municipalities of the WHO/UNICEF Care for Child Development (CCD) methodology. Challenges in understanding and applying the CCD methodology in the context of home visits emerged in 2017 when the first training efforts started and are still happening.<br><br>Monitoring information systems: KIs reported challenges measuring fidelity and quality due to the lack of a friendly monitoring system, cumbersome paper-based forms and tools, and lack of implementation process indicators. Municipal-level supervisors are responsible for monitoring the quality of home visits. This data is used by municipality, state, and federal-level program leaders.	Short introductory/refresher virtual training on the CCD as a strategy to increase training capacity, enhance quality, and reach more home visitors. High number of home visitors who failed the knowledge test.<br><br>Short-cut adaptations were made to the original training protocol to maintain the fast pace of scaling up, for example, an introductory 8 h on the CCD and home visit methodology instead of the 80 h of initial training required (40 h on CCD to strengthen bonding and stimulate development plus 40 h about the home visiting methodology—a stepby- step approach to carry out home visits). This strategy foresaw providing continuing education to complement home visitor training hours. This is especially critical in the context of high turnover of home visitors and supervisors due to an inefficient hiring process coupled with short-term contracts and low salaries. Short introductory/refresher virtual training on the CCD was cited as a strategy to increase training capacity, enhance quality, and reach more home visitors. KIs reported that home visitors appreciated the virtual training. However, in the first virtual training on CCD, KIs were surprised by the high number.<br><br>Delivery: Context-specific adaptations to facilitate the operationalization of PCF according to the municipal characteristics were cited by KIs, including delivery of home visiting through existing community health worker programs to increase dissemination, sustainability, and delivery of PCF intervention at a health center facility, providing both individual and group sessions.	As of December 2020, the program has been implemented in 2934 of Brazil's 5570 municipalities and served 862,000 vulnerable children and 190,8000 pregnant women.<br><br>Barriers to providing adequate care were the strong identification and lack of coordination of home visits for pregnant women and child development with existing healthcare services, and lack of guidance from the federal level on how to conduct the intervention for disabled children and children not living with their families due to social protection measures. Regardless of these challenges, because of the demand by states and municipalities, the federal leadership expanded the eligibility criteria of PCF. Before, only families served by the conditional cash transfer program Bolsa Familia were eligible, but currently any family in social vulnerability registered in the National Database of Vulnerable populations (CADÚNICO) is eligible.<br><br>Barriers to delivering home visits, such as lack of institutional infrastructure and long distances, have affected the ability of municipalities to achieve adequate number and length of home visits for each family.	Sustainability is currently an issue for CF because of strong negative feedback loops that hinder its effectiveness.<br><br>Institutionalization as a national policy is critical to guarantee a continued and stable budget for key programing aspects, such as the training cascade, staff contracts, and appropriate salaries, to prevent high personnel turnover, and decentralized monitoring and evaluation systems. Therefore, political support is considered critical for PCF maintenance.<br><br>A potential enabling strategy for PCF sustainability is to strengthen the bottom-up approach, that is, federal guidance should account for municipal adaptations and specific local contextual factors and demands.<br><br>Critical role of research and evaluation to demonstrate PCF effectiveness.	PCF lacks cofunding from states and municipalities, unlike other national sustainable programs in Brazil, such as the Community Health Workers. State-level KIs reported the following barriers related to funding: (1) shortage of personnel in the state coordination body to provide technical assistance to municipalities, and (2) challenges in operationalizing funding for training due to the bureaucratic administrative processes required to rent a venue for training workshops, for example (PD). Barriers for monthly transfers to municipalities were difficulty in complying with the required number of visits per family, because either the family could not receive the team or other institutional barriers (e.g., lack of transportation) and challenges in registering the activities in the SUAS monitoring information system.	CF emerged at the end of the presidential term that had been born out of impeachment. The volatile political climate added pressure to the scaling up of the program — administrators wanted to ensure that it would attain widespread coverage quickly, thus increasing the odds that the next presidential administration would keep it running. This compromised the quality of the services that CF was able to deliver. In response to this, the federal branch asked for a three-month long strategic pause to prioritize quality rather than volume of scale-up.<br><br>States' roles enabled the rapid scale-up of PCF by speeding up dissemination from federal level to municipalities through seminars and monitoring implementation, by providing supervision and technical assistance to municipalities	Not reported
**Eastern Mediterranean Region**												
**Pakistan** (low-middle income)	Pakistan Early Child Development Scale-up Study (PEDs)	Caregivers: Families with socioeconomic status are at higher risk of ECD delays. This program saw a lower decline in cognitive development over time among low SES families as expected due to the intervention. Attention should be given to helping all families including low SES families to integrate and apply CCD lessons such as by thinking about using accessible materials. A major barrier to participation was caregiver lack of time. It was appropriate that female caregivers did not have to ask elders or partners for permission to participate in the group sessions in most cases. A picture book was provided to each households for its appropriateness due to its widespread value to children of various ages, covering a variety of stimulation activities. While the intervention delivery included children with disabilities, the program could be adapted to support a complex range of disabilities. Program success in the context was dependent on flexibility in training and support and ability to adapt to issues such as power failure and need for training on specific topics. Group meetings were appropriate and inclusive with some families of children with disabilities participating.	PEDS demonstrated the feasibility of integrating nutritional supplementation and CCD into an existing national community health worker program. The use of community health workers facilitated program delivery and additional training and supervision were successfully incorporated into ongoing activities. Early challenges included weak community health worker skills, the difficulty of recruiting women to be community health workers from rural areas, non-compliance of some community health workers, and community skepticism. These were addressed by supportive supervision, appealing content, and a practical approach to skill building. community health workers are not all meeting work requirements within each home visit, so it was feasible to integrate responsive caregiving activities within the time not being used for other things. This intervention used lower frequency CCD visits (from fortnightly or weekly to monthly) which is more feasible. The potential diminished effectiveness because of lower intensity is compensated by using community groups, focusing on skill building, focusing on capacity building, employing effective supervision strategies. Environmental challenges such as heat and power failures discouraged group meetings, so a combined delivery approach addressed this. Group meetings were feasible platforms for community health workers to use their time efficiently.	Caregivers: Caregivers were open to the group meetings and found that they afforded them the opportunity to interact socially, "chit chat", and learn from one another. Caregivers gave feedback that the coverage of the program was not adequate because some households in their community did not get invited to participate, and the intervention should more actively involve other caregivers such as grandmothers. One father described how the home sessions were good because it allowed him opportunities to ask questions about caregiving that aren't acceptable to ask in group settings. Home visits were also acceptable because it was easier for caregivers to attend and make time for in their busy schedule. Once caregivers understood the intervention is for the benefit of their children, they actively participated and accepted the combined interventions. Caregivers reported that there is important spillover encouraging the involvement of other caregivers with mothers encouraging their partners to be active. Additionally, it has spillover on the other siblings who may be older whose parents apply intervention learnings to their care as well. Caregivers were satisfied upon noticing improvements in their child's development, such as meeting developmental milestones. The use of the community health worker to deliver CCD is acceptable to families due to the use of community health workers who have a peer status and can navigate local customs and norms.	Caregivers: 31% of female caregivers participated in monthly group visits and 75% of households received monthly home visits. There was low adoption of nutrition supplements by families who did not give powder to their children. Need better communication about micronutrient powder. Overall, there was a low drop out rate of participating caregivers. Challenges to adoption and participation did occur during a religious period of fasting and a flood emergency, during which group participation was reduced.<br><br>The community participated and integrated activities into their daily activities thanks to a "virtuous" cycle of community satisfaction driving community health worker motivation driving better service delivery, leading to visible results and increasing community satisfaction. Over the course of the intervention, community members bevan trusting the community health worker more and began turning to the community health workers for help.	Workforce: ECD facilitators completed monthly supervisory checklists of community health worker assessing skills such as counseling, nutrition, feedback, and recommendations. Additional mechanisms used to measure fidelity include contact records of home visits, group meetings, and supervision, and training reports. 89-99% of households reported receiving home visits. These revealed a graduate improvement of skills and coaching techniques of community health workers. In Jan 2010, 69% of community health workers praised caregivers whereas in July 2011 91% of community health workers did so. Training and supervision is critical to high quality and fidelity.<br><br>Group meetings ocurred as expected. Program quality drove community buy in and participation.	Intervention adaptation and delivery were designed after a period of formative research identifying gaps in interventions that could improve ECD outcomes. Adaptations include using local illustrations in the communication guide, creating a resource kit with play materials made from local materials, creating a problem solving checklist.<br>During early implementation, it was difficult for community health workers to reach all caregivers with the monthly home visits, so in the early months they put on twice monthly group sessions.	Families: Within the participating catchement areas, 42% of eligible children under the age of 2 received the intervention and the enhanced intervention reached one third of female primary caregivers in the catchment areas.	Sustainability and constant improvement was driven by real-time monitoring at all levels that led to immediate decision making. Record keeping tools included checklist to observe intervention delivery and a record of meetings between supervisors and health workers. Families reported general emotional wellbeing of caregivers which is important for sustainability of activities and effect.<br><br>The supervisors would contact the implementation agents at least twice a month with at least one contact being a site visit where she would conduct a supervisory checklist.	A cost effectiveness trial demonstrated that at 12 months the combined intervention was most cost effective and at 24 months the stand-alone CCD interventione as the most cost effective (likely due to attrition of community health workers in the combined group). ECD facilitators have a critical role in ensuring effectiveness and thus implementation cost effectiveness. The cost of integrating responsive caregiving into existing community health worker program is US 4.00 per child per month. When scaled up, this cost would be driven down as the trial duplicated some supervisory roles that would be integrated into existing supervision costs. The cost of each lady health worker annually, including compensation and support costs, is US $570.	Scaling up would see lower costs as supervision would be integrated into existing supervisor's roles. It would be critical to strengthen trainers and supervisors, perhaps by hiring master trainers at the provincial level. Key scalable components are its low-intensity (only monthly visits), but high-quality programming and the high uptake and motivation of participating families. Specifically, policy makers must pay attention to supervision and training. Continued scale up will depend on using the existing ECD infrastructure and willingness of local program leaders. Data monitoring and reporting will also ensure quality as it goes to scale. ECD indicators have been built into the MoH's district health information software to track newborns with birth defects and children under the age of 5 suspected of ECD delays.	Children receiving the responsive caregiving had higher cognitive, language, and motor development outcomes. The most at risk children (girls, children in poor households, mothers with no education, mothers with psych distress, stunted children, children in food insecure households) who received the CCD intervention improved developmentally to match and exceed not at risk children who did not receive CCD. Demonstrating that this intervention benefits at-risk children and can close the gap. There was no significant difference on child growth from any treatment arm. Integrating nutrition intervention did not have an additive effect on development outcomes. Both intervention groups saw reduced incidence of morbidity. The intervention groups saw decreased prevalence of maternal psychological distress. Investigators determined that maternal depressive symptoms affected longitudinal height for age scores via diarrheal illness. Both treatments improved mother child interaction quality which was associated with improved height for age.
	Sustainable Program Incorporating Nutrition and Games (SPRING)	Caregivers: Program should build on the formative research identifying aspiration as a powerful tool; mothers are overworked and need support; nutrition interventions should focus on first breastfeeding; there is pressure to stop breastfeeding if milk is thought to insufficient; need consistent weaning message; play is already happening in households during daily activities; fathers and other caregivers sometimes play wit the child but this should be strengthened; there are some reports of families using homemade toys, but this is an opportunity; parents need to actively play with their child and observe the benefit. The maternal psychosocial wellbeing addition to the program is appropriate to meet the needs including high stress, negative life events. Remaining barriers include low family support causing women to be overwhelmed with tasks and have little time to interact with their children. While community health worker's had low influence in changing instances of extreme poverty and low education, the use of picture books was appropriate for the population.<br><br>Workforce: The addition of the 5 pillars is designed to facilitate the work of the community health workers, not add to it, so it is appropriate. After an initial investment of time, community health workers are more equipped to address the community issues they see in client families.	Not reported	Workforce: community health worker reported that the thinking healthy program (THP) training helped them deliver all messages more effectively as health educators. (pg 110). Health workers found the family engagement strategy effective, they felt like they had tools to engage the family and felt ownership of the program. community health workers found that the problem solving tasks gave them tools to be useful.<br>Caregivers: Mothers found the empathetic listening of the community health workers helpful. Mothers found the use of pictures interesting and engaging. Breaking down steps in behavioral actiation was effective and suitable to the caregivers. Families did not have high acceptability of the intervention as they did not find that it provided new methods of caregiving compared to how they already played with their children. They wanted more guidance on how their child could self-play. Caregvers were unwilling to repeat activities if they believed they weren't helping and did not think all activities were age appropriate.	Not reported	Adaptation following the feasibility trial. Program implementers developed the five-pillar approach to maternal psychosocial well-being to integrate into the program: (1) family support: during initial home visit community health workers spent time engaging key decision makers including mothers-in-laws and husbands, (2) empathetic listening: sessions begin open-ending with community health workers listening to the caregivers' free talk (3) guided discovery: health messages are conveyed using pictures, (4) behavioral activation: community health worker helps caregiver break down behavior change into achievable steps, (5) Problem solving: community health worker visits previous problems and goals brought up in the last session. This improved the appropriateness and effectiveness of the intervention.	Not reported	Not reported	Strong supervision and refresher training is necessary to maintain quality and sustained effect.	Not reported	Integrating the maternal psychosocial program into the existing community health worker and SPRING programs demonstrates an effective way to scale up a maternal metnal health program. Additionally, using the five pillars in other without using the CBT program is still an effective way to improve maternal mental health.	differences in effect size between intervention and control groups were small and mostly not statistically significant at 18 months.
**Europe Region**												
**Turkey** (upper-middle income)	Care for Development	The intervention was only delivered once as it is difficult to reach children where there is not a strong system to provide continuous well-child care.	Adding the intervention into the existing pediatric visit was feasible and did not lower the quality of the clinical services.	Workforce: the trained pediatricians felt more confident in their relationship with patients and families following the CDI training.	Not reported	Not reported	Not reported	Not reported	Not reported	Not reported	Not reported	Families who received the CDI had higher levels of play in the home, made toys more often, and caregivers more frequently read to their child yielded an overall more optimal home environment for ECD.
**Kazakhstan** (low-middle income)<br>**Tajikistan** (low income)<br>**Kyrgyz Republic** (low income)	Better Parenting Initiative (Kazakhstan), IMCI with CCD (Tajikistan, Kyrgyz Republic)	Kazakhstan, Tajikistan, Kyrgyz Republic:<br>CCD improved likelihood of mothers trying new activities with their children but did not improve rates of fathers playing with their children. Compared to older children, younger children has more improved development in the group receiving CCD.<br><br>Kyrgyz Republic: Challenges to caregivers implementing CCD activities include low comprehension of the lessons and the lack of materials.	Not reported	Kazakhstan, Tajikistan, Kyrgyz Republic:<br>Caregivers: mothers and other caregivers were satisfied with the care they received from staff trained in IMCI and IMCI with CCD.<br>Workforce: There is a high demand for additional training in CCD from health providers.<br><br>Kyrgyz Republic:<br>Workforce: Over half of all family doctors requested additional CCD training.	Not reported	The quality of the program varies greatly among clinical settings with trained CCD providers.	A common recommendation provided during CCD education was to massage the child, following a cultural traditional of massage.	Not reported	Kazakhstan, Tajikistan, Kyrgyz Republic:<br>While some child development indicators were tracked by health providers, there was no strong supervisory or monitoring system, impeding the program's sustainable implementation.<br><br>Kazakhstan: A facilitator of sustainability was the institutionalization of CCD. CCD became a part of the required package of early child health servies.	Not reported	Not reported	Kazakhstan, Tajikistan, Kyrgyz Republic:<br>Healthcare workers trained in CCD felt more preapred to provide care to young children and better able to use the Mother's card. Trained providers were also more confident to provide nutrition education. In the trained group, providers were more likely to give recommendations about CCD.<br><br>Tajikistan, Kyrgyz Republic:<br>Children receiving CCD had improved communitcation, gross motor, and personal social development
**South-East Asia Region**												
**India** (middle income)	Project Grow Smart	Caregivers: The program was adapted to better meet caregiver needs by incorporating feedback from mothers in the pilot study into future training of the home visitors. The common questions posed by mothers in the pilot became part of an FAQ lesson used in the training to equip home visitors to better meet caregiver needs. The pilot test revealed that mothers did not always remember the key messages so a reinforcing booster session was added to the intervention. Some mothers reported feedback that the intervention might be more useful to less educated, illiterate mothers.<br>Workforce: Some home visitors had low confidence when conducting visits with mothers who were more educated than them or mothers who were from a higher social class. Initially visitors just read the instructions and had a difficult time explaining the importance of early learning. This was addressed by adapting the training and integrating ongoing learning into regular supervision meetings. Supervisors praise visitors when they used active listening techniques.	The pilot program was implemented effectively and was acceptable to caregivers. The supervision and training was functional. The pilot study's feasibility provided justification for the full RCT.	Caregivers: Mothers emphasized their desire for their children to grow up smart and healthy which led the team to name the project "Project Grow Smart." The initial pilot test revealed the need to change some of the language of the intervention. The use of "cognitive" was changed to "smart" and "food texture" was changed to "food variety." The team also dropped an activity using mirrors because mothers reported the widespread belief that that would delay speech. The lead interventionist conducted an survey of the participants and found that mothers were satisfied and enthusiastic about the program. They believed the program helped their children become smarter, even saying their younger children became more healthy and smart than their older children. Mothers were most receptive to the intervention when they noticed changes in their child's development. Some did not understand activities like banging pots when they did not understand the rationale.	In the infant phase, the village-level worker supervisors recruit and enroll the participants and supervise and monitor the implementation of the intervention.	Supervisors reviewed the home visitors records of the visits and attended a home visit once monthly to conduct an observation record. Supervisors accompany home visitors on monthly visits where the complete an observation record to monitor the quality. The Home-Visit Evaluation Form is completed by the village-level worker after every contact to address participant compliance and questions/concerns, and note village-level worker advice/encouragement. Home-Visit Evaluation Forms are reviewed by supervisors weekly to verify fidelity and to address any concerns raised by mothers or the village-level worker. Supervisors complete an Observation Record checklist following the monthly home visit observations; the record is to assesses the quality of the interactions between the village-level worker and the mother. They review these checklists during supervision to improve intervention quality.<br><br>Supervisors are trained over one month on operating procedures, completing monitoring forms, and general support techniques. They are provided with training and operation manuals, which are prepared by the investigators (and include job descriptions and standard operating procedures, including instructions on the completion of monitoring forms and the FAQ). The training is completed over 1 month and includes practice role plays and completion of monitoring forms.	CCD material and material from the PEDS study was adapted the context by using colorful pictures of locally representative children and caregivers in an adapted flipchart for use during the visits. Local phrases, songs, and play activities were incorporated into the intervention. The visitors also used an already popular children's book during the visit.	Not reported	Supervisors meet with individual village-level workers every other week to help problem solve, replenish product supplies, review participant progress, and to discuss problems/solutions in increase program success related to scheulding and intervention visits.	Not reported	Not reported	Children who received CCD alone or in combination with nutrition supplementation had improved visual receptive and expressive language performance scores.
	Sustainable Program Incorporating Nutrition and Games (SPRING)	Caregivers: Formative research identified key ECD needs and opportunities including: aspiration as a powerful tool; mothers are overworked and need support; nutrition interventions should focus on first breastfeeding; there is pressure to stop breastfeeding if milk is thought to insufficient; need consistent weaning message; play is already happening in households during daily activities; fathers and other caregivers sometimes play with the child but this should be strengthened; there are some reports of families using homemade toys, but this is an opportunity; parents need to actively play with their child and observe the benefit. The SPRING intervention fits these needs by modifying caregiver-child interactions that are critical in preventing exposure to childhood adversity that leads to poor ECD.	Not reported	Caregivers: Key design choices were built on formative research to make the program acceptable. There was an emphasis on breastfeeding to fill the need gap, emphasis on complementary foods and high-protein foods, play messages integrated into program, the entire family was integrated into the program delivery. Families did not have high acceptability of the intervention as they did not find that it provided new methods of caregiving compared to how they already played with their children. They wanted more guidance on how their child could self-play. Caregivers were unwilling to repeat activities if they believed they weren't helping and did not think all activities were age appropriate.<br>Workforce: community health worker were recruited from the local area and were empathetic and patient. community health workers found that the problem solving tasks gave them tools to be useful.	Not reported	Individual supervision consists of tracking quality and performance, giving individual feedback, and occurs in the field. The group supervision addresses common issues and challenges, provides peer group learning, and is conducted in the health center.	Not reported	Not reported	The delivery mode of the intervention was feasible and determined to be sustainable in the context of India's existing ECD services.	Using a new cadres of community-based home visitors instead of highly trained specialists kept implementation costs low	Not reported	differences in effect size between intervention and control groups were small and mostly not statistically significant at 18 months. Potential reasons include family time restraints preventing care givers from practicing responsive caregiving, the perception that the intervention did not offer anything new, and the low engagement of non-mother caregivers such as grandmothers.
**Western Pacific Region**												
**Vietnam** (low-middle income)	Learning Clubs	Caregivers: A visual-based package was used in order to be appropriate for low-literacy populations. Fathers and grandmothers are also encouraged to be involved. The pilot test revealed that the number of modules in the curriculum was too large and unweild and so was reduced from 24 to 20 sessions.	The feasibility of conducting group interventions was established by the pilot test. The platform of group sessions was efficient at reaching a greater number of caregivers.	Caregivers: content was highly acceptable to participants who reported higher confidence in caregiving and satisfaction with the new knowledge.<br>Workforce: Facilitators were confident in providing the content but expressed a need for more training on mental health as it is a new concept to them. They also expressed a desire for more training in toy construction.	Caregivers: There was incredibly high interest in the program leading to Learning Club attendance far exceeding the goals. At the end of the pilot test 918 people had attended with 305 of those being partners or grandparents.	The facilitator's guide used by the implementor ensures fidelity.	Material: The curriculum was made by integrating CCD, the Thinking Healthy Program, and other child health programs adapted to the local context in Vietnam. The materials were field tested which determined their salience and comprehensibility. In order to ensure acceptability, the translation of original English material into Vietnamese was done by multiple bilingual research assistants. The material was also augmented with illustrations by a Vietnamese graphic designer.	Not reported	Not reported	Included in the list of prorgam-related costs were: training and transportation operation, facilitator manuals, training courses for provincial trainers, and training courses for commune facilitators.	Not reported	Not reported
**China** (low-middle, upper-middle income)	IMCI with CCD	Caregivers: 'Mother's Cards' were adapted for China from the WHO global CFD materials. All mothers received good communicatoin demonstrations and practice opportunities, using the Mother's Card as a reference point to help them remember the verbal advice. This was considered particularly suitable for mothers with low literacy and/or education.	Professionals stressed that mothers could use household items and materials (ie pots, wooden utensils, pegs, clothes, bottles, stones). Mothers also reported increased feasibility of activities with continued experience of counseling and practicing of the recommendations. Results suggested that the implementation of these guidelines was feasible for rural households.	Not reported	Not reported	Workforce: Implementers delivered CCD with fidelity using language and communication skills as taught within WHO's IMCI package.	Materials: 'Mother's Cards' were adapted for China from the WHO global CFD materials. The card showed age-specific messages for promoting effective play and communication between caregiver and child. The pictures use clear line drawings, simple language. All mothers received good communicatoin demonstrations and practice opportunities, using the Mother's Card as a reference point to help them remember the verbal advice. This was considered particularly suitable for mothers with low literacy or education.	Not reported	Not reported	Not reported	Not reported	Results suggest that the two counseling sessions were associated with better short-term developmental outcomes, especially for cognitive, language, and social dimensions. A larger indoor living space was associated with better motor scores. The most consistent correlations were related to the nature of child-caregiver interactions. More than 90% of mothers who received the intervention began playing more purposefully with their children on a daily basis, including games like cup stacking, number counting, and making cardboard jigsaws.
	Integrated Early Childhood Development Programme (IECD)	Counselling services were relatively passive, meaning families lacked the active follow-up or promotion efforts needed to reinforce the effects of promoting child-feeding behaviors at home. This study found the significant intervention effect on the developmental outcome only for ages 12-24 months, which further underlining the importance of consistent and early exposure with sufficient time for the intervention.	The ECD center, a fixed working station, was effective for this study working with a large population.	Not reported	Not reported	Not reported	Not reported	Not reported	Community service capacities must be strengthened in the poor, rural areas in order to integrate new services into existing prgrams to be cost-effective.<br><br>Need to better leverage the strength of the existing, well-established health systems in the country to work together to provide ECD services to children under age 3.	Not reported	Not reported	Prevalence of overall suspected developmental delay was reduced by 18% in intervention counties (from 37% at baseline to 19% at post-intervention) compared with the 10% reduction seen in control counties. Consistent findings were found across domains and analysis indicated that the effect of the intervention on promoting developmental health was mediated by multiple nurturing care-associated factors, such as cognitive stimulation frequency, positive discipline, length-for-age growth and haemoglobin concentration.

[illegible]
Not reported
The passage of the 2018 Siaya County Health Services Bill marks the first time that ECD content has been included in a health bill in Kenya, providing a strong regulatory framework to anchor health programs that embed developmental monitoring and support for nurturing care.
Not reported
In Mozambique, PATH's advocacy efforts resulted in the inclusion of early childhood development content in the pre-service training curricula of nurses and community health workers, which are now under revision.
Not reported
Not reported
Not reported
Not reported
Not reported
Not reported
Not reported
Not reported
Not reported
Not reported
Not reported
Not reported
