# Supplementary material for: Pathways to scale up early childhood programs: A scoping review of Reach Up and Care for Child Development
Source: PLOS Glob Public Health. 2023 Aug 9;3(8):e0001542. doi: 10.1371/journal.pgph.0001542 (PMC10411826; doi:10.1371/journal.pgph.0001542)
Supplement: S8 Table — (PDF) [file pgph.0001542.s010.pdf]

**S6 Table. Implementation Strategies Screening by Building**

| Implementation Strategies | Program Emergence | | | | | Intersectoriality | | | | | Intervention Characteristics | | | | | | | | Workforce | | | | | | | Training | | | | | | Monitoring System | | | | | |
|---|---|---|---|---|---|---|---|---|---|---|---|---|---|---|---|---|---|---|---|---|---|---|---|---|---|---|---|---|---|---|---|---|---|---|---|---|---|
| | Governance and Leadership | Multilevel Networks | Funding | Scaling Process | Integration into ECD system | Coordination and Communication | Intervention Characteristics | Intersectoral Targeting | Intersectoral Workforce | Delivery Sites and Networks | Intervention Delivery | Intervention Setting | Dose and Frequency | Design Approach | Materials | Caregiver Engagement | Supplementary program activities | Responsive Teaching | Workforce recruitment | Implementation Agent Training | Job Satisfaction | Compensation and Contracts | Caseload | Workforce Characteristics | Community Integration | Training manuals and protocols | Training Skills | Continuing Education | Training Leadership | Training Design | Institutionalization | Supportive Mentoring | Standardized Data Collection Tools | Data-Informed Decision Making | Supervisor Training | Supervisor Caseload | Impact Measurement |
| **CCD** | | | | | | | | | | | | | | | | | | | | | | | | | | | | | | | | | | | | | |
| **African Region** | | | | | | | | | | | | | | | | | | | | | | | | | | | | | | | | | | | | | |
| Kenya: Smart Start Siaya County | Yes | Yes | Yes | Yes | Yes | Yes | Yes | No | Yes | Yes | Yes | Yes | Not identified | Yes | Yes | Yes | No | Not identified | Yes | Yes | Yes | Yes | Not identified | Yes | Yes | Not identified | Not identified | Yes | Yes | Yes | Yes | Yes | Yes | Yes | Yes | Not identified | Not identified |
| Kenya: Msingi Bora | Yes | Yes | Yes | Yes | Yes | Not identified | Yes | No | Yes | Not identified | Yes | Yes | Yes | Yes | Yes | Yes | Yes | Yes | Yes | Yes | Not identified | Yes | Not identified | Not identified | Yes | Yes | Not identified | Yes | Yes | Yes | No | Yes | Yes | Yes | Yes | Not identified | Yes |
| Malawi: CCD | Yes | Yes | Yes | Yes | No | Yes | Yes | No | Yes | Yes | Yes | Yes | Yes | Yes | Yes | Yes | Yes | Yes | Yes | Yes | No | Yes | Yes | Yes | Yes | Yes | Not identified | Not identified | No | Yes | No | Not identified | Yes | Not identified | Yes | Not identified | Yes |
| Mozambique: Nurturing Care Collaboration | Yes | Yes | Yes | No | Yes | Yes | Yes | No | Yes | Yes | Yes | Yes | Yes | Yes | Yes | Not identified | Yes | Not identified | Yes | Yes | Not identified | Not identified | Not identified | Not identified | Yes | Yes | Yes | Yes | Yes | Yes | Yes | Yes | Yes | Yes | Yes | Not identified | Yes |
| Rwanda: Sugira Muryango | Yes | Yes | Yes | Yes | No | Not identified | Yes | Yes | No | No | Yes | Yes | Yes | Yes | Not identified | Yes | Yes | Yes | Yes | Yes | Not identified | Yes | Not identified | Yes | Yes | Yes | Yes | Not identified | Yes | Yes | No | Yes | Yes | Yes | Yes | Not identified | Yes |
| Tanzania: Integrated health, nutrition, responsive stimulation package | Yes | Not identified | Not identified | No | Not identified | Not identified | Yes | Yes | No | Yes | Yes | Yes | Yes | Yes | Not identified | Yes | Yes | Yes | Yes | Yes | Not identified | Yes | Not identified | Yes | Not identified | Yes | Yes | Not identified | Yes | Yes | No | Yes | Yes | Yes | Not identified | Not identified | Yes |
| **Americas Region** | | | | | | | | | | | | | | | | | | | | | | | | | | | | | | | | | | | | | |
| Brazil: Criança Feliz | Yes | Yes | Yes | Yes | Yes | Yes | No | Yes | No | No | Yes | Yes | Yes | Yes | Yes | Not identified | No | Not identified | Yes | Yes | Not identified | Yes | Yes | Not identified | Not identified | Yes | Yes | Yes | Yes | Yes | Yes | Yes | Yes | Yes | Not identified | Yes | Yes |
| **Eastern Mediterranean Region** | | | | | | | | | | | | | | | | | | | | | | | | | | | | | | | | | | | | | |
| Pakistan: PEDS | Not identified | Yes | Yes | Yes | Yes | Yes | Yes | No | Yes | Not identified | Yes | Yes | Yes | Yes | Yes | Yes | Yes | Yes | Yes | Yes | Not identified | Yes | Yes | Yes | Yes | Not identified | Yes | Yes | Yes | Yes | No | Yes | Yes | Yes | Yes | Yes | Yes |
| Pakistan: SPRING | Not identified | Yes | Yes | Yes | Yes | Yes | Yes | No | Yes | No | Yes | Yes | Not identified | Yes | Not identified | Yes | Yes | Yes | Yes | Yes | Not identified | Not identified | Not identified | Yes | Yes | Not identified | Yes | Yes | Not identified | Yes | No | Yes | Not identified | Not identified | Yes | Yes | Yes |
| **Europe Region** | | | | | | | | | | | | | | | | | | | | | | | | | | | | | | | | | | | | | |
| Turkey: Care for Development | No | No | Yes | No | Yes | Not identified | Yes | Yes | Yes | Yes | Yes | Yes | Yes | Yes | Yes | No | No | No | Yes | Yes | Yes | Yes | Yes | No | No | Not identified | Not identified | Not identified | Not identified | Yes | No | Not identified | Not identified | Not identified | Not identified | Not identified | Yes |
| Kazakhstan: Better Parenting Initiative | Yes | Yes | Yes | Yes | Yes | Yes | Yes | No | Yes | Yes | Yes | Yes | Not identified | Yes | Yes | Not identified | Not identified | Not identified | Yes | Yes | Not identified | Not identified | Not identified | Not identified | Yes | Yes | Not identified | Yes | Yes | Yes | Yes | Not identified | Yes | Not identified | Not identified | Not identified | Yes |
| Tajikistan: IMCI with CCD | Yes | Yes | Yes | Yes | Yes | Yes | Yes | No | Yes | Yes | Yes | Yes | Not identified | Yes | Yes | Not identified | Not identified | Not identified | Yes | Yes | Not identified | Not identified | Not identified | Not identified | Yes | Yes | Yes | Not identified | No | Yes | Yes | Not identified | Yes | Not identified | Not identified | Not identified | Yes |
| Kyrgyz Republic: IMCI with CCD | Yes | Yes | Yes | Yes | Yes | Yes | Yes | No | Yes | Yes | Yes | Yes | Not identified | Yes | Yes | Not identified | Not identified | Not identified | Yes | Yes | Not identified | Not identified | Not identified | Not identified | Yes | Yes | Yes | Yes | Not identified | Yes | Yes | Not identified | Yes | Not identified | Not identified | Not identified | Yes |
| **South-East Asia Region** | | | | | | | | | | | | | | | | | | | | | | | | | | | | | | | | | | | | | |
| India: SPRING | Yes | Yes | Yes | No | Yes | Not identified | Yes | No | Yes | No | Yes | Yes | Not identified | Yes | Yes | Not identified | Yes | Not identified | Yes | Yes | Yes | Not identified | Not identified | Yes | Yes | Not identified | Not identified | Yes | No | Yes | No | Yes | Yes | Yes | Not identified | Not identified | Yes |
| India: Project Grow Smart | Yes | Yes | Yes | No | No | Not identified | Yes | No | No | No | Yes | Yes | Yes | Yes | Yes | Not identified | Yes | Yes | Yes | Yes | Yes | Yes | Yes | Yes | Yes | Yes | Yes | Yes | Not identified | Yes | Not identified | Yes | Yes | Yes | Yes | Yes | Yes |
| **Western Pacific Region** | | | | | | | | | | | | | | | | | | | | | | | | | | | | | | | | | | | | | |
| Vietnam: Learning Clubs | Not identified | Yes | Yes | Yes | Yes | Not identified | Yes | No | Yes | Yes | Yes | Yes | Not identified | Yes | Yes | Yes | Yes | Not identified | Yes | Yes | Not identified | Not identified | Not identified | Not identified | Yes | Yes | Not identified | Not identified | Yes | Yes | No | Not identified | Yes | Not identified | Yes | Not identified | Yes |
| China: IMCI | Not identified | Not identified | Yes | Yes | No | Not identified | No | No | Not identified | Not identified | Not identified | Not identified | Yes | Yes | Yes | Not identified | Yes | Yes | Not identified | Yes | Not identified | Not identified | Not identified | Not identified | Not identified | Not identified | Yes | Not identified | Not identified | Not identified | Not identified | Not identified | Yes | Not identified | Not identified | Not identified | Yes |
| China: IECD | Not identified | Yes | Yes | Yes | Yes | Yes | Yes | No | Yes | Yes | Yes | Yes | Yes | Yes | Yes | Not identified | Yes | Not identified | Yes | Yes | Not identified | Not identified | Not identified | Not identified | Not identified | Not identified | Not identified | Not identified | Not identified | Not identified | Not identified | Yes | Yes | Not identified | Not identified | Not identified | Yes |
| | | | | | | | | | | | | | | | | | | | | | | | | | | | | | | | | | | | | | |
| **African Region** | | | | | | | | | | | | | | | | | | | | | | | | | | | | | | | | | | | | | |
| Zimbabwe: Modified RU | Not identified | Not identified | Yes | Not identified | Yes | Not identified | No | Not identified | Yes | Yes | Yes | Yes | Yes | Not identified | Yes | Not identified | No | Not identified | Yes | Yes | Yes | Not identified | Not identified | Not identified | Not identified | Yes | No | Yes | Not identified | Yes | Not identified | Yes | Yes | Yes | Yes | Yes | Not identified |
| Madagascar: Early Stimulation | Yes | Yes | Yes | No | Yes | Not identified | Yes | No | No | No | Yes | Yes | Not identified | Yes | Yes* | Not identified | Yes | Not identified | Yes | Yes | Not identified | Not identified | Not identified | Yes | Not identified | Not identified | Not identified | Yes | No | Yes | No | Yes | Yes | Not identified | Not identifed | Not identified | Yes |
| **Americas Region** | | | | | | | | | | | | | | | | | | | | | | | | | | | | | | | | | | | | | |
| Brazil: Responsive Caregiving and Early Learning Program | Yes | Yes | Yes | Yes | Yes | Not identified | No | Yes | Yes | No | Yes | Yes | Yes | Yes | Yes | Yes | No | Not identified | Yes | Yes | Not identified | Yes | Not identified | Yes | Not identified | Yes | Not identified | Yes | Yes | Yes | No | Yes | Yes | Yes | Yes | Not identified | Not identified |
| Peru: Cuna Más | Yes | Yes | Yes | Yes | Yes | Yes | No | Yes | Yes | No* | Yes | Yes | Yes | Yes | Yes | Not identified | No | Not identified | Yes | Yes | Yes | Yes | Yes | Yes | Yes | Yes | Yes | Yes | Yes | Yes | Yes | Yes | Yes | Yes | Yes | Yes | Yes* |
| Colombia: Home:Based ECD Intervention | Yes | Yes | Yes | Yes | Yes | Not identified | Yes | Yes | No | No | Yes | Yes | Not identified | Yes | Yes | Not identified | Yes | Not identified | Yes | Yes | Yes | Yes* | Yes | Yes | Yes | Yes | Not identified | Yes | Not identified | Yes | No | Yes | Yes | Yes | Yes | Yes | Yes* |
| Colombia: Enhanced FAMI | Yes | Yes | Yes | Yes | Yes | Not identified | Yes | Not identified | No | Yes | Yes | Yes | Yes | Yes | Yes | Yes | Yes | Yes | Yes | Yes | Yes | Not identified | Not identified | Yes | Yes | Yes | Yes | Not identified | Not identified | Yes | No | Yes | Yes | Not identified | Not identified | Not identified | Yes |
| Jamaica: JHVP | No | No | Yes | No | No | Not identified | Yes | No | Yes | No | Yes | Yes | Yes | Yes | Yes | No | Yes | Yes | Yes | Yes | Not identified | Not identified | Not identified | Not identified | Not identified | Yes | Yes | Not identified | Yes | Yes | No | Yes | Yes | Yes | Yes | Not identified | Yes |
| **Eastern Mediterranean Region** | | | | | | | | | | | | | | | | | | | | | | | | | | | | | | | | | | | | | |
| Jordan: Modified Reach Up and Learn | Yes | Yes | Yes | Not identified | Yes | Yes | No | No | No | No | Yes | Yes | Yes | Yes | Yes | Yes | No | Yes | Yes | Yes | Yes | Yes | Yes | Yes | Yes | Yes | Yes | Not identified | Yes | Yes | Not identified | Yes | Yes | Yes | Yes | Yes | Yes |
| Lebanon: Modified Reach Up and Learn | Yes | Yes | Yes | Not identified | Yes | Yes | No | No | No | No | Yes | Yes | Yes | Yes | Yes | Yes | No | Yes | Yes | Yes | Yes | Yes | Yes | Yes | Yes | Yes | Yes | Not identified | Yes | Yes | Not identified | Yes | Yes | Yes | Yes | Yes | Yes |
| Syria: Modified Reach Up and Learn | Yes | Yes | Yes | Not identified | Yes | Yes | No | No | No | No | Yes | Yes | Yes | Yes | Yes | Yes | No | Yes | Yes | Yes | Yes | Yes | Yes | Yes | Yes | Yes | Yes | Not identified | Yes | Yes | Not identified | Yes | Yes | Yes | Yes | Yes | Yes |
| **Western Pacific Region** | | | | | | | | | | | | | | | | | | | | | | | | | | | | | | | | | | | | | |
| Bangladesh: Integrated psychosocial stimulation and UCT | Yes | Yes | Yes | No | No | Not identified | Yes | Yes | No | No | Yes | Yes | Yes | Yes | Yes | Yes | Yes | Yes | Yes | Yes | Not identified | No | Yes | Yes | No | Yes | Not identified | Not identified | Yes | Yes | Not idenfitied | Yes | Yes | Not identified | Not identified | Not identified | Yes |
| Bangladesh: RINEW | Yes | Yes | Yes | No | No | Not identified | Yes | Yes | No | No | Yes | Yes | Yes | Yes | Yes | Yes | Yes | Yes | Yes | Yes | Not identified | No | Yes | Yes | No | Yes | Not identified | Yes | Not identified | Yes | Not idenfitied | Yes | Yes | Yes | Not identified | Yes | Yes |
| Bangladesh: RU-modified | Yes | Yes | Yes | Yes | Yes | Not identified | Yes | Yes | Yes | Yes | Yes | Yes | Yes | Yes | Yes | Not identified | Yes | Not identified | Yes | Yes | Not identified | Yes | Yes | Yes | Not identified | Yes | Yes | Yes | Not identified | Yes | Not idenfitied | Yes | Yes | Yes | Yes | Not identified | Yes |
| Bangladesh: Psychosocial Stimulation | Not identified | Yes | Yes | Yes | Yes | Not identified | Yes | Yes | No | Yes | Yes | Yes | Yes | Yes | Yes | Not identified | Yes | Not identified | Yes | Not identified | Not identified | Not identified | Not identified | Yes | Yes | Not identified | Not identified | Not identified | Not identified | Not idenfitied | Not idenfitied | Not identified | Yes | Not identified | Not identified | Not identified | Yes |
| Bangladesh: Jamaica-modified | Yes | Yes | Yes | Yes | Yes | Not identified | Yes | Yes | No | Yes | Yes | Yes | Yes | Yes | Yes | Not identified | Yes | Not identified | Yes | Yes | Not identified | Not identified | Not identified | Yes | Not identified | Not identified | Not identified | Not idenfitied | Not identified | Not idenfitied | Not idenfitied | Yes | Yes | Yes | Not identified | Not identified | Yes |
| **Total number of programs** | | | | | | | | | | | | | | | | | | | | | | | | | | | | | | | | | | | | | |
| Yes | 24 | 28 | 32 | 20 | 25 | 14 | 25 | 12 | 18 | 15 | 32 | 32 | 24 | 32 | 30 | 15 | 21 | 15 | 32 | 32 | 11 | 16 | 13 | 21 | 20 | 23 | 17 | 17 | 16 | 29 | 7 | 25 | 31 | 20 | 18 | 11 | 30 |
| No | 9 | 5 | 1 | 13 | 8 | 19 | 8 | 21 | 15 | 18 | 1 | 1 | 9 | 1 | 3 | 18 | 12 | 18 | 1 | 1 | 22 | 17 | 20 | 12 | 13 | 10 | 16 | 16 | 17 | 4 | 26 | 8 | 2 | 13 | 15 | 22 | 3 |
| Not identified | 7 | 3 | 1 | 4 | 1 | 19 | 0 | 2 | 1 | 3 | 1 | 1 | 9 | 1 | 3 | 16 | 3 | 17 | 1 | 1 | 21 | 15 | 20 | 11 | 10 | 10 | 15 | 15 | 13 | 2 | 7 | 8 | 2 | 13 | 14 | 22 | 3 |
