# Supplementary material for: Pathways to scale up early childhood programs: A scoping review of Reach Up and Care for Child Development
Source: PLOS Glob Public Health. 2023 Aug 9;3(8):e0001542. doi: 10.1371/journal.pgph.0001542 (PMC10411826; doi:10.1371/journal.pgph.0001542)
Supplement: S10 Table — (DOCX) [file pgph.0001542.s012.docx]

| **S8a. List of Included Records for Care for Child Development programs** | | | | |
| --- | --- | --- | --- | --- |
| # | Country (Program Name) | Citation | |  |
| African Region | | | | |
| 1 | Kenya (Smart Start Siaya County) |  | Friedman, L., & Wolfheim, C. (2019). Landscape Analysis of Survive, Thrive, and Transform Interventions for Children. USAID. https://www.healthynewbornnetwork.org/hnn-content/uploads/Landscape-Analysis-of-Survive.pdf |  |
|  |  |  | Putcha, V., Bonsu, D., Hatipoglu, K., Neuman, M., & Roland, M. (2019). Country Brief, Kenya: Empowering Community Health Volunteers to Integrate Nurturing Care: Offering Ongoing Support and Better Recognition early childhood workforce initiative. The Early Childhood Workforce Initiative. https://www.r4d.org/wp-content/uploads/Brief-Kenya.pdf |  |
|  |  | * | World Health Organization, United Nations Children’s Fund, World Bank Group. (2018). Nurturing care for early childhood development: a framework for helping children survive and thrive to transform health and human potential. (Licence: CC BY-NC-SA 3.0 IGO.) World Health Organization. https://apps.who.int/iris/bitstream/handle/10665/272603/9789241514064-eng.pdf |  |
| 2 | Kenya (Msingi Bora) |  | Luoto, J. E., Lopez Garcia, I., Aboud, F. E., Fernald, L., & Singla, D. R. (2019). Testing means to scale early childhood development interventions in rural Kenya: the Msingi Bora cluster randomized controlled trial study design and protocol. BMC public health, 19(1), 259. https://doi.org/10.1186/s12889-019-6584-9 |  |
|  |  |  | Luoto, J. E., Garcia, I. L., Aboud, F. E., Singla, D. R., Fernald, L. C. H., Pitchik, H. O., Saya, U. Y., Otieno, R., & Alu, E. (2021). Group-based parenting interventions to promote child development in rural Kenya: A multi-arm, cluster-randomised community effectiveness trial. The Lancet Global Health, 9(3), e309–e319. https://doi.org/10.1016/S2214-109X(20)30469-1 |  |
| 3 | Malawi (Care for Child Development) |  | Gladstone, M., Phuka, J., Mirdamadi, S., Chidzalo, K., Chitimbe, F., Koenraads, M., & Maleta, K. (2018). The care, stimulation and nutrition of children from 0-2 in Malawi-Perspectives from caregivers; "Who's holding the baby?". PloS one, 13(6), e0199757. https://doi.org/10.1371/journal.pone.0199757 |  |
|  |  |  | Phuka, J., Maleta, K., Thomas, M., & Gladstone, M. (2014). A job analysis of community health workers in the context of integrated nutrition and early child development. Annals of the New York Academy of Sciences, 1308, 183–191. https://doi.org/10.1111/nyas.12338 |  |
|  |  |  | Gladstone, M., Phuka, J., Thindwa, R., Chitimbe, F., Chidzalo, K., Chandna, J., Gleadow Ware, S. and Maleta, K. (2018), Care for Child Development in rural Malawi: a model feasibility and pilot study. Ann. N.Y. Acad. Sci., 1419: 102-119. https://doi.org/10.1111/nyas.1372 |  |
| 4 | Mozambique (Nurturing Care Collaboration) |  | Gray, K., Frey, M., & Sen, D. (2018). Reaching the youngest children: an approach for health system integration. Early Childhood Matters, 127, 43-47. https://bernardvanleer.org/app/uploads/2018/06/ECM18_ENG.pdf |  |
|  |  |  | Rodrigues, M., Kaneko, M., Bermudez, C., Lombardi, J., Fisher, S., & Berelowitz, D. (2019). Scaling Up Place-Based Strategies to Strengthen Community Early Childhood Systems. Spring Impact. https://www.springimpact.org/wp-content/uploads/2019/12/Scaling up-place-based-strategies_Dec2019_FINAL_WEB.pdf |  |
|  |  | * | Mitter, R. & Putcha, V. (2018). Strengthening and Supporting the Early Childhood Workforce: Training and Professional Development. Early Childhood Workforce Initiative. https://www.r4d.org/wp-content/uploads/Training_Professional_Development_ExecutiveSummary.pdf |  |
|  |  |  | Jeong J., Bliznashka, L., Ahun, M.N., Karuskina-Drivdale, S.K. et al. (2022). A pilot to promote early childhood development within health systems in Mozambique: a qualitative study. Ann. N.Y. Acad. Sci. doi:10.111/nyas.14718 |  |
| 5 | Rwanda: Sugira Muryango | * | Arriagada, A.-M., Perry, J., Rawlings, L., Trias, J., & Zumaeta, M. (2018). Promoting Early Childhood Development through Combining Cash Transfers and Parenting Programs. World Bank, Washington, DC. https://doi.org/10.1596/1813-9450-8670 |  |
|  |  |  | Barnhart, D.A., Farrar, J., Murray, S.M. et al. Lay-worker Delivered Home Visiting Promotes Early Childhood Development and Reduces Violence in Rwanda: A Randomized Pilot. J Child Fam Stud 29, 1804–1817 (2020). https://doi.org/10.1007/s10826-020-01709-1 |  |
|  |  |  | Betancourt, T.S., Jensen, S.K.G., Barnhart, D.A. et al. Promoting parent-child relationships and preventing violence via home-visiting: a pre-post cluster randomised trial among Rwandan families linked to social protection programmes. BMC Public Health 20, 621 (2020). https://doi.org/10.1186/s12889-020-08693-7 |  |
|  |  |  | Betancourt, T. S., Franchett, E., Kirk, C. M., Brennan, R. T., Rawlings, L., Wilson, B., Yousafzai, A., Wilder, R., Mukunzi, S., Mukandanga, J., Ukundineza, C., Godfrey, K., & Sezibera, V. (2020). Integrating social protection and early childhood development: Open trial of a family home-visiting intervention, Sugira Muryango. Early Child Development and Care, 190(2), 219–235. https://doi.org/10.1080/03004430.2018.1464002 |  |
| 6 | Tanzania: integrated health, nutrition, responsive stimulation package |  | Sudfeld, C.R., Bliznashka, L., Ashery G., Yousafzai, A.K., Masanja H (2019). Effect of a community health worker delivered health, nutrition and responsive stimulation package and conditional cash transfers on child development and growth in rural Tanzania: protocol for a cluster-randomized trial BMC Public Health (2019) 19:641 https://doi.org/10.1186/s12889-019-7008-6 |  |
|  |  |  | Bliznashka, L. Yousafzai, A.K., Ashery G., Masanja H., Sudfeld C. (2021) Effects of a community health worker delivered intervention on maternal depressive symptoms in rural Tanzania. Health Policy and Planning, 36. doi:10.1093/heapol/czaa170 |  |
| Americas Region | | | | |
| 7 | Brazil (Criança Feliz) |  | Halim Antonio Girade, H. A. (2018). ‘Criança Feliz’: A programme to break the cycle of poverty and reduce the inequality in Brazil. Early Childhood Matters, Bernard VanLeer Foundation. 34-38. https://bernardvanleer.org/app/uploads/2018/06/2.1-Crianc%CC%A7a-Feliz.pdf |  |
|  |  |  | Buccini, G., Venancio, S. I., & Pérez‐Escamilla, R. (2021). Scaling up of Brazil’s Criança Feliz early childhood development program: An implementation science analysis. Annals of the New York Academy of Sciences. https://doi.org/10.1111/nyas.14589 |  |
|  |  |  | Sokolovic, N., Schneider, A., Perlman, M., Souza, R., Jenkins, J., M. (2022). Teaching home-visitors to support responsive caregiving: a cluster randomized controlled trial of an online professional development program in Brazil. Journal of Global Health, 12, doi:10.7189/jogh.12.04007 |  |
| Eastern Mediterranean Region | | | | |
| 8 | Pakistan (Early Child Development Scale-up Study [PEDs]) | * | Mitter, R. & Putcha, V. (2018). Strengthening and Supporting the Early Childhood Workforce: Training and Professional Development. Early Childhood Workforce Initiative. https://www.r4d.org/wp-content/uploads/Training_Professional_Development_ExecutiveSummary.pdf |  |
|  |  |  | Brown, N., Finch, J. E., Obradović, J., & Yousafzai, A. K. (2017). Maternal care mediates the effects of nutrition and responsive stimulation interventions on young children's growth. Child: care, health and development, 43(4), 577–587. https://doi.org/10.1111/cch.12466 |  |
|  |  |  | Gowani, S., Yousafzai, A. K., Armstrong, R., & Bhutta, Z. A. (2014). Cost effectiveness of responsive stimulation and nutrition interventions on early child development outcomes in Pakistan. Annals of the New York Academy of Sciences, 1308, 149–161. https://doi.org/10.1111/nyas.12367 |  |
|  |  |  | Lucas, J. E., Richter, L. M., & Daelmans, B. (2018). Care for Child Development: an intervention in support of responsive caregiving and early child development. Child: care, health and development, 44(1), 41–49. https://doi.org/10.1111/cch.12544 |  |
|  |  |  | Yousafzai, A. K., Rasheed, M. A., Rizvi, A., Armstrong, R., & Bhutta, Z. A. (2014). Effect of integrated responsive stimulation and nutrition interventions in the Lady Health Worker programme in Pakistan on child development, growth, and health outcomes: A cluster-randomised factorial effectiveness trial. The Lancet, 384(9950), 1282–1293. https://doi.org/10.1016/S0140-6736(14)60455-4 |  |
|  |  |  | Yousafzai, A. K., Rasheed, M. A., & Siyal, S. (2018). Integration of parenting and nutrition interventions in a community health program in Pakistan: an implementation evaluation. Annals of the New York Academy of Sciences, 1419(1), 160–178. https://doi.org/10.1111/nyas.13649 |  |
|  |  |  | Petrovic, O., & Yousafzai, A. (2013). Promoting Care for Child Development in Community Health Services: A Summary of the Pakistan Early Child Development Scale-up (PEDS) Trial: Main findings, delivery strengths ain findings, delivery strengths and the path forward. UNICEF. https://sites.unicef.org/earlychildhood/files/3_PEDS_Trial_Summary_Report.pdf |  |
|  |  |  | Yousafzai, A. K., Rasheed, M. A., Rizvi, A., Armstrong, R., & Bhutta, Z. A. (2015). Parenting Skills and Emotional Availability: An RCT. Pediatrics, 135(5), e1247–e1257. https://doi.org/10.1542/peds.2014-2335 |  |
| 9 | Pakistan (SPRING) |  | Zafar, S., Sikander, S., Haq, Z., Hill, Z., Lingam, R., Skordis-Worrall, J., Hafeez, A., Kirkwood, B., & Rahman, A. (2014). Integrating maternal psychosocial well-being into a child-development intervention: the five-pillars approach. Annals of the New York Academy of Sciences, 1308, 107–117. https://doi.org/10.1111/nyas.12339 |  |
|  |  | * | World Health Organization, United Nations Children’s Fund, World Bank Group. (2018). Nurturing care for early childhood development: a framework for helping children survive and thrive to transform health and human potential. (Licence: CC BY-NC-SA 3.0 IGO.) World Health Organization. https://apps.who.int/iris/bitstream/handle/10665/272603/9789241514064-eng.pdf |  |
| Europe Region | | | | |
| 10 | Turkey (Care for Development) |  | Ertem, I. O., Atay, G., Bingoler, B. E., Dogan, D. G., Bayhan, A., & Sarica, D. (2006). Promoting Child Development at Sick-Child Visits: A Controlled Trial. Pediatrics, 118(1), e124. https://doi.org/10.1542/peds.2005-2704 |  |
| 11 | Kazakhstan (Better Parenting Initiative) |  | Engle, P., Najimidinova, G., & Faromuzova, K. (2011). Care for development in three Central Asian countries: Report of a process evaluation in Tajikistan, Kyrgyz Republic, and Kazakhstan. UNICEF. https://citeseerx.ist.psu.edu/viewdoc/download?doi=10.1.1.434.850&rep=rep1&type=pdf |  |
| 12 | Tajikistan (IMCI with CCD) |  |  |  |
| 13 | Kyrgyz Republic (IMCI with CCD) |  |  |  |
| South-East Asia Region | | | | |
| 14 | India (SPRING) |  | Bhopal, S., Roy, R., Verma, D., Kumar, D., Avan, B., Khan, B., Gram, L., Sharma, K., Amenga-Etego, S., Panchal, S. N., Soremekun, S., Divan, G., & Kirkwood, B. R. (2019). Impact of adversity on early childhood growth & development in rural India: Findings from the early life stress sub-study of the SPRING cluster randomised controlled trial (SPRING-ELS). PloS one, 14(1), e0209122. https://doi.org/10.1371/journal.pone.0209122 |  |
|  |  |  | Boucheron, P., Bhopal, S., Verma, D., Roy, R., Kumar, D., Divan, G., & Kirkwood, B. (2020). Observed feeding behaviours and effects on child weight and length at 12 months of age: Findings from the SPRING cluster-randomized controlled trial in rural India. PloS one, 15(8), e0237226. https://doi.org/10.1371/journal.pone.0237226 |  |
|  |  |  | Kilkaari Program: Booklet to support home visits. (n.d.). Sangath. https://spring.lshtm.ac.uk/files/2016/09/Kilkari-Manual_2016_English.pdf |  |
|  |  |  | Divan, G., Bondre, A., Hill, Z., Lingam R., Singhal, N., Hooda, N., Sareen, R. l., Panchal, A., Skordis Worral, J., Rahman, A., & Kirkwood, B. (n.d.). SPRING Kilkaari: Counseling Aids for Promotion of ECD in Haryana, India. Sangath. https://spring.lshtm.ac.uk/files/2016/10/SPRING-Counselling-Aid-Poster-Final.pdf |  |
|  |  |  | Lingam, R., Gupta, P., Zafar, S., Hill, Z., Yousafzai, A., Iyengar, S., Sikander, S., Haq, Z. u., Mehta, S., Skordis-Worrel, J., Rahman, A., & Kirkwood, B. (2014). Understanding care and feeding practices: building blocks for a sustainable intervention in India and Pakistan. Annals of the New York Academy of Sciences, 1308, 204–217. https://doi.org/10.1111/nyas.12326 |  |
|  |  | * | World Health Organization, United Nations Children’s Fund, World Bank Group. (2018). Nurturing care for early childhood development: a framework for helping children survive and thrive to transform health and human potential. (Licence: CC BY-NC-SA 3.0 IGO.) World Health Organization. https://apps.who.int/iris/bitstream/handle/10665/272603/9789241514064-eng.pdf |  |
| 15 | India (Project Grow Smart) |  | Hurley, K., Fernandez-Rao, S., Nair, K. M., Balakrishna, N., Radhakrishna, K., Ravinder, P., Tilton, N., Reinhart, G., Harding, K., & Black, M. (2019). Grow Smart: An Integrated Nutrition and Early Child Development Intervention Among Infants Improves Expressive Language and Reduces Anemia and Iron Deficiency in Rural India (FS08-03-19). Current Developments in Nutrition, 3(Supplement_1). https://doi.org/10.1093/cdn/nzz044.FS08-03-19 |  |
|  |  |  | Fernandez-Rao, S., Hurley, K. M., Nair, K. M., Balakrishna, N., Radhakrishna, K. V., Ravinder, P., Tilton, N., Harding, K. B., Reinhart, G. A., & Black, M. M. (2014). Integrating nutrition and early child-development interventions among infants and preschoolers in rural India. Annals of the New York Academy of Sciences, 1308, 218–231. https://doi.org/10.1111/nyas.12278 |  |
| Western Pacific Region | | | | |
| 16 | Vietnam (Learning Clubs) | * | World Health Organization, United Nations Children’s Fund, World Bank Group. (2018). Nurturing care for early childhood development: a framework for helping children survive and thrive to transform health and human potential. (Licence: CC BY-NC-SA 3.0 IGO.) World Health Organization. https://apps.who.int/iris/bitstream/handle/10665/272603/9789241514064-eng.pdf |  |
|  |  |  | Nguyen, T., Sweeny, K., Tran, T., Luchters, S., Hipgrave, D. B., Hanieh, S., Tran, T., Tran, H., Biggs, B. A., & Fisher, J. (2019). Protocol for an economic evaluation alongside a cluster randomised controlled trial: cost-effectiveness of Learning Clubs, a multicomponent intervention to improve women's health and infant's health and development in Vietnam. BMJ open, 9(12), e031721. https://doi.org/10.1136/bmjopen-2019-031721 |  |
|  |  |  | Learning clubs for women’s health and infant development. (n.d.). Saving Brains. Retrieved June 17, 2021, from https://www.savingbrainsinnovation.net/projects/0344-03/ |  |
|  |  |  | Fisher, J., Tran, T., Luchters, S., Tran, T. D., Hipgrave, D. B., Hanieh, S., Tran, H., Simpson, J., Nguyen, T., Le, M., & Biggs, B.-A. (2018). Addressing multiple modifiable risks through structured community-based Learning Clubs to improve maternal and infant health and infant development in rural Vietnam: Protocol for a parallel group cluster randomised controlled trial. BMJ Open, 8(7), e023539. https://doi.org/10.1136/bmjopen-2018-023539 |  |
| 17 | China (IMCI with CCD) |  | Walker, S. P., Wachs, T. D., Grantham-McGregor, S., Black, M. M., Nelson, C. A., Huffman, S. L., Baker-Henningham, H., Chang, S. M., Hamadani, J. D., Lozoff, B., Gardner, J. M., Powell, C. A., Rahman, A., & Richter, L. (2011). Inequality in early childhood: risk and protective factors for early child development. Lancet (London, England), 378(9799), 1325–1338. https://doi.org/10.1016/S0140-6736(11)60555-2 |  |
|  |  |  | Jin, X., Sun, Y., Jiang, F., Ma, J., Morgan, C., & Shen, X. (2007). "Care for Development" intervention in rural China: a prospective follow-up study. Journal of developmental and behavioral pediatrics : JDBP, 28(3), 213–218. https://doi.org/10.1097/dbp.0b013e31802d410b |  |
| 18 | China (Integrated Early Childhood Development Programme [IECD]) |  | Zhou, S., Zhao, C., Huang, X., Li, Z., Ye, R., Shi, H., Zhao, Q., Zhou, Y., Chen, X., O'Sullivan, M., Pouwels, R., Martin, K., Zhang, J., Wang, X., & Scherpbier, R. W. (2019). The effect of a community-based, integrated and nurturing care intervention on early childhood development in rural China. Public health, 167, 125–135. https://doi.org/10.1016/j.puhe.2018.11.010 |  |
| *Citation included information from more than one program | | | | |
|  | | | | |
| **S8b. List of Included Records for Reach Up programs** | | | | |
| # | Country (Program Name) | Citation | |  |
| African Region | | | | |
| 1 | Zimbabwe (Modified Reach Up) | * | Smith, J. A., Baker-Henningham, H., Brentani, A., Mugweni, R., & Walker, S. P. (2018). Implementation of Reach Up early childhood parenting program: acceptability, appropriateness, and feasibility in Brazil and Zimbabwe. Annals of the New York Academy of Sciences, 1419(1), 120–140. https://doi.org/10.1111/nyas.13678 |  |
| 2 | Madagascar (Early Stimulation) |  | Galasso, E., Weber, A. M., Stewart, C. P., Ratsifandrihamanana, L., & Fernald, L. (2019). Effects of nutritional supplementation and home visiting on growth and development in young children in Madagascar: a cluster-randomised controlled trial. The Lancet. Global health, 7(9), e1257–e1268. https://doi.org/10.1016/S2214-109X(19)30317-1 |  |
| Americas Region | | | | |
| 3 | Brazil (Responsive Caregiving and Early Learning Program) | * | Smith, J. A., Baker-Henningham, H., Brentani, A., Mugweni, R., & Walker, S. P. (2018). Implementation of Reach Up early childhood parenting program: acceptability, appropriateness, and feasibility in Brazil and Zimbabwe. Annals of the New York Academy of Sciences, 1419(1), 120–140. https://doi.org/10.1111/nyas.13678 |  |
|  |  | * | Kohli-Lynch, M., Ponce Hardy, V., Bernal Salazar, R., Bhopal, S. S., Brentani, A., Cavallera, V., Goh, E., Hamadani, J. D., Hughes, R., Manji, K., Milner, K. M., Radner, J., Sharma, S., Silver, K. L., Lawn, J. E., & Tann, C. J. (2020). Human resources and curricula content for early child development implementation: multicountry mixed methods evaluation. BMJ open, 10(4), e032134. https://doi.org/10.1136/bmjopen-2019-032134 |  |
|  |  | * | Milner, K. M., Bernal Salazar, R., Bhopal, S., Brentani, A., Britto, P. R., Dua, T., Gladstone, M., Goh, E., Hamadani, J., Hughes, R., Kirkwood, B., Kohli-Lynch, M., Manji, K., Ponce Hardy, V., Radner, J., Rasheed, M. A., Sharma, S., Silver, K. L., Tann, C., & Lawn, J. E. (2019). Contextual design choices and partnerships for scaling early child development programmes. Archives of Disease in Childhood, 104(Suppl 1), S3. https://doi.org/10.1136/archdischild-2018-315433 |  |
| 4 | Peru: Cuna Más | * | Arriagada, A.-M., Perry, J., Rawlings, L., Trias, J., & Zumaeta, M. (2018). Promoting Early Childhood Development through Combining Cash Transfers and Parenting Programs. World Bank, Washington, DC. https://doi.org/10.1596/1813-9450-8670 |  |
|  |  |  | Early Childhood Workforce Initiative. (2017). Supporting the Early Childhood Workforce at Scale: The Cuna Más home visiting program in Peru. http://repositorio.minedu.gob.pe/bitstream/handle/20.500.12799/5528/Supporting%20the%20early%20childhood%20workforce%20at%20scale%20The%20Cuna%20M%c3%a1s%20home%20visiting%20program%20in%20Peru.pdf?sequence=1&isAllowed=y |  |
| 5 | Colombia (Home-based Early Childhood Development Intervention) |  | Andrew, A., Attanasio, O., Fitzsimons, E., Grantham-McGregor, S., Meghir, C., & Rubio-Codina, M. (2018). Impacts 2 years after a scalable early childhood development intervention to increase psychosocial stimulation in the home: A follow-up of a cluster randomised controlled trial in Colombia. PLOS Medicine, 15(4), e1002556. https://doi.org/10.1371/journal.pmed.1002556 |  |
|  |  |  | Attanasio, O., UCL(2010). Colombia Medium-term Effects of Home-based Early Childhood Development Intervention Impact Evaluation (MICS) 2010, Baseline Survey. (Ref. COL_2010_ECDIIE-BL_v01_M.). https://microdata.worldbank.org/index.php/catalog/3405/study-description |  |
|  |  |  | Attanasio, O. P., Fernández, C., Fitzsimons, E. O. A., Grantham-McGregor, S. M., Meghir, C., & Rubio-Codina, M. (2014). Using the infrastructure of a conditional cash transfer program to deliver a scalable integrated early child development program in Colombia: Cluster randomized controlled trial. BMJ, 349, g5785. https://doi.org/10.1136/bmj.g5785 |  |
|  |  |  | Attanasio, O., Cattan, S., Fitzsimons, E., Meghir, C., & Rubio-Codina, M. (2015). Estimating the Production Function for Human Capital: Results from a Randomized Control Trial in Colombia (No. w20965). National Bureau of Economic Research. https://doi.org/10.3386/w20965 |  |
|  |  |  | Berlinski, S., & Schady, N. (2015). More Bang for the Buck: Investing in Early Childhood Development. In S. Berlinski & N. Schady (Eds.), The Early Years (pp. 149–178). Palgrave Macmillan US. https://doi.org/10.1057/9781137536495_6 |  |
|  |  |  | World Bank. (2019). Colombia: Can a Successful Parenting Program be Implemented at Scale? (English). (139102). From Evidence to Policy note series Washington, D.C.: World Bank Group. http://documents1.worldbank.org/curated/en/822611562924879162/pdf/Colombia-Can-a-Successful-Parenting-Program-be-Implemented-at-Scale.pdf |  |
|  |  | * | Arriagada, A.-M., Perry, J., Rawlings, L., Trias, J., & Zumaeta, M. (2018). Promoting Early Childhood Development through Combining Cash Transfers and Parenting Programs. World Bank, Washington, DC. https://doi.org/10.1596/1813-9450-8670 |  |
| 6 | Colombia: Enhanced FAMI | * | Kohli-Lynch, M., Ponce Hardy, V., Bernal Salazar, R., Bhopal, S. S., Brentani, A., Cavallera, V., Goh, E., Hamadani, J. D., Hughes, R., Manji, K., Milner, K. M., Radner, J., Sharma, S., Silver, K. L., Lawn, J. E., & Tann, C. J. (2020). Human resources and curricula content for early child development implementation: multicountry mixed methods evaluation. BMJ open, 10(4), e032134. https://doi.org/10.1136/bmjopen-2019-032134 |  |
|  |  | * | Milner, K. M., Bernal Salazar, R., Bhopal, S., Brentani, A., Britto, P. R., Dua, T., Gladstone, M., Goh, E., Hamadani, J., Hughes, R., Kirkwood, B., Kohli-Lynch, M., Manji, K., Ponce Hardy, V., Radner, J., Rasheed, M. A., Sharma, S., Silver, K. L., Tann, C., & Lawn, J. E. (2019). Contextual design choices and partnerships for scaling early child development programmes. Archives of Disease in Childhood, 104(Suppl 1), S3. https://doi.org/10.1136/archdischild-2018-315433 |  |
|  |  |  | Gomez, M.L., Bernal R., Baker-Henningham H. (2022). Qualitative evaluation of a scalable early childhood parenting programme in rural Colombia. Child Care Health Dev., 48:225-238. doi:10.1111/cch.12921 |  |
| 7 | Jamaica (Jamaican Home Visiting Program) |  | Gertler, P., Heckman, J., Pinto, R., Zanolini, A., Vermeersch, C., Walker, S., Chang, S. M., & Grantham-McGregor, S. (2014). Labor market returns to an early childhood stimulation intervention in Jamaica. Science (New York, N.Y.), 344(6187), 998–1001. https://doi.org/10.1126/science.1251178 |  |
|  |  |  | Grantham-McGregor, S., & Walker, S. (2015). The Jamaican early childhood home visiting intervention. Early Childhood Matters. https://bernardvanleer.org/app/uploads/2017/10/5.-The-Jamaican-early-childhood.pdf |  |
|  |  |  | Walker, S. P., Chang, S. M., Smith, J. A., & Baker-Henningham, H. (2018). The Reach up Early Childhood Parenting Program: Origins, Content, and Implementation. ZERO TO THREE, 38(4), 37–43. |  |
|  |  |  | Grantham-McGregor, S. M., Walker, S. P., Chang, S. M., & Powell, C. A. (1997). Effects of early childhood supplementation with and without stimulation on later development in stunted Jamaican children. The American journal of clinical nutrition, 66(2), 247–253. https://doi.org/10.1093/ajcn/66.2.247 |  |
|  |  |  | Walker, S. P., Chang, S. M., Powell, C. A., & Grantham-McGregor, S. M. (2005). Effects of early childhood psychosocial stimulation and nutritional supplementation on cognition and education in growth-stunted Jamaican children: prospective cohort study. Lancet (London, England), 366(9499), 1804–1807. https://doi.org/10.1016/S0140-6736(05)67574-5 |  |
|  |  |  | Powell, C., Baker-Henningham, H., Walker, S., Gernay, J., & Grantham-McGregor, S. (2004). Feasibility of integrating early stimulation into primary care for undernourished Jamaican children: Cluster randomised controlled trial. BMJ, 329(7457), 89. https://doi.org/10.1136/bmj.38132.503472.7C |  |
|  |  |  | Gardner, J. M., Powell, C. A., Baker-Henningham, H., Walker, S. P., Cole, T. J., & Grantham-McGregor, S. M. (2005). Zinc supplementation and psychosocial stimulation: effects on the development of undernourished Jamaican children. The American journal of clinical nutrition, 82(2), 399–405. https://doi.org/10.1093/ajcn.82.2.399 |  |
|  |  |  | Grantham-McGregor, S. M., Fernald, L. C., Kagawa, R. M., & Walker, S. (2014). Effects of integrated child development and nutrition interventions on child development and nutritional status. Annals of the New York Academy of Sciences, 1308, 11–32. https://doi.org/10.1111/nyas.12284 |  |
| Eastern Mediterranean Region | | | | |
| 8 | Syria (Modified Reach Up and Learn) |  | Vachon, A. & Wiltonneed, K. (2020). Reach Up and Learn in the Syria Response: Adapting and implementing an evidence-based home visiting program in Lebanon, Jordan and Syria. International Rescue Committee. https://reliefweb.int/sites/reliefweb.int/files/resources/irc-rul-reportapril27-2020.pdf |  |
| 9 | Lebanon (Modified Reach Up and Learn) |  |  |  |
| 10 | Jordan (Modified Reach Up and Learn) |  |  |  |
| Western Pacific Region | | | | |
| 11 | Bangladesh (Modified Jamaican Home Visiting Program) |  | Hamadani, J. D., Huda, S. N., Khatun, F., & Grantham-McGregor, S. M. (2006). Psychosocial stimulation improves the development of undernourished children in rural Bangladesh. The Journal of nutrition, 136(10), 2645–2652. https://doi.org/10.1093/jn/136.10.2645 |  |
| 12 | Bangladesh (Psychosocial Stimulation) |  | Nahar, B., Hossain, M. I., Hamadani, J. D., Ahmed, T., Huda, S. N., Grantham-McGregor, S. M., & Persson, L. A. (2012). Effects of a community-based approach of food and psychosocial stimulation on growth and development of severely malnourished children in Bangladesh: a randomised trial. European journal of clinical nutrition, 66(6), 701–709. https://doi.org/10.1038/ejcn.2012.13 |  |
| 13 | Bangladesh (Modified Reach Up) |  | Hamadani, J. D., Mehrin, S. F., Tofail, F., Hasan, M. I., Huda, S. N., Baker-Henningham, H., Ridout, D., & Grantham-McGregor, S. (2019). Integrating an early childhood development programme into Bangladeshi primary health-care services: an open-label, cluster-randomised controlled trial. The Lancet. Global health, 7(3), e366–e375. https://doi.org/10.1016/S2214-109X(18)30535-7 |  |
|  |  | * | Kohli-Lynch, M., Ponce Hardy, V., Bernal Salazar, R., Bhopal, S. S., Brentani, A., Cavallera, V., Goh, E., Hamadani, J. D., Hughes, R., Manji, K., Milner, K. M., Radner, J., Sharma, S., Silver, K. L., Lawn, J. E., & Tann, C. J. (2020). Human resources and curricula content for early child development implementation: multicountry mixed methods evaluation. BMJ open, 10(4), e032134. https://doi.org/10.1136/bmjopen-2019-032134 |  |
|  |  | * | Milner, K. M., Bernal Salazar, R., Bhopal, S., Brentani, A., Britto, P. R., Dua, T., Gladstone, M., Goh, E., Hamadani, J., Hughes, R., Kirkwood, B., Kohli-Lynch, M., Manji, K., Ponce Hardy, V., Radner, J., Rasheed, M. A., Sharma, S., Silver, K. L., Tann, C., & Lawn, J. E. (2019). Contextual design choices and partnerships for scaling early child development programmes. Archives of Disease in Childhood, 104(Suppl 1), S3. https://doi.org/10.1136/archdischild-2018-315433 |  |
|  |  |  | Mehrin SF, Hamadani JD, Salveen NE, Hasan MI, Hossain SJ, Baker-Henningham H. Adapting an Evidence-Based, Early Childhood Parenting Programme for Integration into Government Primary Health Care Services in Rural Bangladesh. Front Public Health. 2021 Jan 18;8:608173. doi: 10.3389/fpubh.2020.608173. |  |
| 14 | Integrated responsive stimulation, maternal mental health, nutrition, WASH and lead exposure prevention interventions (RINEW) |  | Akter F, Rahman M, Pitchik HO, Winch PJ, Fernald LCH, Nurul Huda TM, Jahir T, Amin R, Das JB, Hossain K, Shoab AK, Khan R, Yeasmin F, Sultana J, Luby SP, Tofail F. Adaptation and Integration of Psychosocial Stimulation, Maternal Mental Health and Nutritional Interventions for Pregnant and Lactating Women in Rural Bangladesh. Int J Environ Res Public Health. 2020 Aug 27;17(17):6233. doi: 10.3390/ijerph17176233. |  |
|  |  |  | Pitchik HO, Tofail F, Rahman M, Akter F, Sultana J, Shoab AK, Huda TMN, Jahir T, Amin MR, Hossain MK, Das JB, Chung EO, Byrd KA, Yeasmin F, Kwong LH, Forsyth JE, Mridha MK, Winch PJ, Luby SP, Fernald LC. A holistic approach to promoting early child development: a cluster randomised trial of a group-based, multicomponent intervention in rural Bangladesh. BMJ Glob Health. 2021 Mar;6(3):e004307. doi: 10.1136/bmjgh-2020-004307. |  |
| 15 | Integrated psychosocial stimulation and unconditional cash transfer |  | Hossain SJ, Roy BR, Salveen NE, Hasan MI, Tipu SMMU, Shiraji S, Tofail F, Hamadani JD. Effects of adding psychosocial stimulation for children of lactating mothers using an unconditional cash transfer platform on neurocognitive behavior of children in rural Bangladesh: protocol for a cluster randomized controlled trial. BMC Psychol. 2019 Mar 5;7(1):13. doi: 10.1186/s40359-019-0289-9. PMID: 30836984; PMCID: PMC6402152. |  |
|  |  |  | Hossain SJ, Roy BR, Sujon HM, Tran T, Fisher J, Tofail F, El Arifeen S, Hamadani JD. Effects of integrated psychosocial stimulation (PS) and Unconditional Cash Transfer (UCT) on Children's development in rural Bangladesh: A cluster randomized controlled trial. Soc Sci Med. 2022 Jan;293:114657. doi: 10.1016/j.socscimed.2021.114657. Epub 2021 Dec 15. PMID: 34942577. |  |
| *Citation included information from more than one program | | | | |
