# Supplementary material for: Pathways to scale up early childhood programs: A scoping review of Reach Up and Care for Child Development
Source: PLOS Glob Public Health. 2023 Aug 9;3(8):e0001542. doi: 10.1371/journal.pgph.0001542 (PMC10411826; doi:10.1371/journal.pgph.0001542)
Supplement: S4 Table — (PDF) [file pgph.0001542.s006.pdf]

S4a Table. Qualitative data collection of implementation strategies of Care for Child Development programs

Country	Program Name	ECD systems	Sectoral leadership and partnership	Funding	Focal Nurturing Care Domains	Intersectoral Approach to Program Collaboration	Intervention Setting (individual or group)	Dose	Frequency	Design Approach (universal, selective, indicated)	Materials	Number of Implementation Agends	Implementation Agent Caseload	Implementation Agent Characteristics and Recruitment	Personal Contracts and Compensation	Training manuals and protocols	Training	Refresher Trainings	Supervisor Characteristics and Recruitment	Supervisor Training	Number of Supervisors	Caseload of Supervisors (number of implementation agents per supervisor)	Workforce Mentorship: support of home visitors	Program Data and Reporting
**African Region**																								
**Kenya** (low income)	Msingi Bora	In 2006, Kenya passed the national Early Childhood Development Policy Framework. In 2010, Kenya introduced a new constitution affording more governing power to counties instead of the central government. Local health infrastructure and ministry partners were motivated to address ECD issues. In 2018, there was a new overarching national policy for newborn, child, and adolescent health. Kenya has made progress in ECD with gains in pre-primary education (77% pre-primary gross enrollment in 2016), but a notable gap exists for services from age 0-3.<br>Msingi Bora: No other significant ECD programs were operating in the rural districts where this program was implemented. The national NGO Safe Water and Aids Project (SWAP) operated three community centers in local towns that were used during implementation.<br>Smart Start Siaya County: Home visits began in 2014 in Siaya county, but there were challenges such as turnover, poor training, lack of resources, no pay, and balancing other employment. In 2015, Siaya county became the first to pay its community health volunteers a stipend.	Partnership between US and Canada based university researchers and the Safe Water and Aids Project.	Research Project	Good Health<br>Adequate nutrition<br>Responsive caregiving	The Msingi Bora curriculum includes responsive caregiving, hygiene, nutrition, and love and respect in the family. It was delivered by community health volunteers who are also tasked with promoting community health and referring patients to primary health facilities.	Home (individual) and outside of home (group)	90 minutes	fortnightly	**Indicated:** Children aged 6-24 months without severe impairment at baseline were eligible; however, only the households contacted were enrolled in order to limit workload and improve feasibility. Villages were eligible if there were at least 20 households with eligible children, one CHV already assigned, and the villages were geographically distributed to prevent crossover. Female caretakers were 15 years old or older, fathers had to be aged 18 and older. Eligibility was determined in collaboration with the Safe Water and Aids Project.	**Play:** role-playing activities, games with materials from the home (e.g. cup, bowl, stones), singing, storytelling	40	Not reported	Each village had a preexisting community health volunteer who was then recruited to participate in this program. No volunteer refused. They are part time volunteers belonging to their community with the main task of improving community health and referring individuals to primary ehalth services. The community health volunteers included 10 men and 30 women with a median age of 44 years, 11 years education, and 9 years of community health volunteer experience.	The program paid community health volunteers a monthly stipend according to a local policy.	Community health workers received session-specific activities and materials, with Luo or Swahili and English manuals.	8 days intensive training covering content for the first 8 sessions. Training was led by study investigators.	8 days intensive training half way through the intervention covering content for the last 8 sessions. This training was led by the Safe Water and Aids Project staff in a train the trainer model	The NGO staff at the local community center who have at least 2 years post-secondary education served as supervisors.	8 days intensive training during which 15 staff were trained and the 3 top performing staff were selected as supervisors while the remaining 12 were specifically trained on data collection.	3	Approximately 13 (calculated)	Supervisors provide feedback to home visitors following sessions. This feedback included comments on skills of facilitating discussion, coaching parents, andwering questions, and overall session quality.	Supervisors track fidelity by using attendance sheets and monitoring checklists during most group village sessions and some individual sessions (in practice, they monitored 89% of group sessions and 9% of individual sessions). Ability of the home visitors was monitoring by assessing the following skills: comments on skills of facilitating discussion, coaching parents, andwering questions, and overall session quality. In addition to the primary supervisors, program data is monitored and analyzed by specific program monitors and an external team of staff from the Kenya Medical Research Institute.
	Smart Start Siaya County (PATH)		Smart Start is driven by leadership of the county governor and first lady. The governor was trained by a Harvard ECD program funded by Hilton Foundation and designed by policy makers at Aga Khan University.	Government and external organizations	Good Health<br>Adequate nutrition<br>Opportunities for early learning<br>Responsive caregiving<br>Security and safety	This program is multi-sectoral with the multi-sectoral team coordinating partnerships between all minisitries. Specifically, CCD is delivered at local clinics where staff also care for child health. Other activities include partnership with the ministry of agriculture on a home garden program promoting nutrition and WASH interventions. The ministry of education is also a close collaborator. Security and safety is addressed by progress in policy reform of the justice system to prevent and mitigate child abuse and neglect.	Home (individual) and outside of home (individual)	Not reported	Not reported	**Universal**: Smart Start is a county wide program integrating nurturing care activities. CCD is delivered to children aged 0 to 3 years.	**Play**: Community health volunteers construct and distribute toys to local families with children.	537	Not reported	Community health volunteers are community members able to read and write, influential community members, or former traditional birth attendants.	Community health volunteers were not paid until 2015 when the governor introduced stipends of $29.30 monthly.	Actors at the county level were trained by private sector and NGO partners to equip key health ministry staff with knowledge of the importance of ECD; they, in turn, helped sensitize frontline health workers to the urgency of ECD action, increasing motivation among staff to deliver the nurturing care program.	Community health assistants are responsible for CHV training. New CHVs go through 10 days of training based on national curriculum delivered by a cascade model. Clinics each have one staff member (i.e. doctor, nurse, technician) trained to train others in CCD	CHVs go through annual continuing education sessions lasting 3-5 days.	Community health assistants who are certified nursing or public health workers are based at health clinics and supervise the CHVs. New supervisors called mentor coordinators were hired to supervise and complement the existing supervisors. Mentor coordinators identified training needs, collecting data and monitor quality, and coaching.	Not reported	Currently, The number of mentor coordinators is not sufficient to support all of the CHVs.	Not reported	Community health assistants and mentor coordinators oversee the community health visitors who deliver CCD, and provide coaching on areas of weak performance. Community health supervisors are responsible for deliving ongoing training messages from the county leadership to the community health visitors.	There are clear reporting mechanisms for community and program data to be used by program implementers and local government. Community health assistants and mentor coordinators identify training needscollect data and deliver it to the multi-sectoral coordination team for use in local decision making.
**Malawi** (low income)	CCD	The Ministry of Gender, Children, and Social Welfare (MoGCSW) and the Ministry of Health (MoH) play a big role in ECD at the community level and both have policies to guide internal implementation of ECD and nutrition activities. The MoH employs health surveillance assistants (HSAs) who deliver routine healthcare and are expected to provide for about 1000 people. Malwi also has national community-based child care centers for children aged 3-5 years but does not provide services for children younger than 3 years of age.<br>Malawi national policy encourages multisectoral collaboration, with comprehensive policies and well-outlined coordination structures for nutrition and ECD that advocate for integrated approaches.	UK and Malawi based researchers partnered with the College Medicine in Malawi, Ministry of Health and Nutrition and solicited input from the Ministry of Gender and Social Welfare, Ministry of Education, Office of the President of the Cabinet for Nutrition, UNICEF, WHO, and Save the Children.	Government and research project	Good Health<br>Adequate nutrition<br>Responsive caregiving	Health Surveillance Assistants (HSAs) delivered the CCD pacakge in addition to nutrition and sanitation related content.	Home (individual) and out of home (group)	Individual 30 minutes; group 45 minutes	Two per month of each group sessions and home visits	**Selective:** the team took a census of the study area to record all children under the age of two at the time of recruitment. All children were entered into a computer generated randomization process and had equal probability of being selected.	**Print Materials:** CCD checklist<br>**Play:** home visitors encouraged to bring example play materials (suhch as spoons, pegs, tins with beans, cloth dolls, cars made out of clay), but not provided with them	6	Ten participants per agent	Implementation agents were recruited from the existing cadre of health surveillance assistants. Health surveillance assistants were often associated with the village clinics that primarily offered health educaiton, growth, monitoring, and dispensing of drugs. Health surveillance assistants were hired by the goverment from anywhere in the country; no longer are they appointed by the community or from the community they serve.	Health surveillance assistants did not receive additional compensation for implementing CCD on top of their existing workload. Country-wide, health surveillance assistants have 100% of their salary covered by the government.	Trained in adapted CCD packaged developed after extensive qualitative formative research and Child's Healthy Growth and Development training materials.	Upon initial hiring, Health Surveillance Assistants received 8-12 weeks of training.The research team conducted a specific training for the Health Surveillance Assistants on the adapted CCD package.	Not reported	Research assistants who were trained on the CCD package supervised implementation.	Research assistants were also trained in the CCD package.	2	3 (calculated)	Not reported	Research assistants monitored delivery quality and fidelity by attending two group and two individual sessions of each of the implementation agents monthly. The recorded data including the implementation agent's general ability, the inclusion of demonatrations, explanations of CCD guidance, praise and encouragement of mothers, and if there was a knowledge check of the caregivers at the end of the session.
**Mozambique** (low income)	Nurturing Care Collaboration (PATH)	In Monapo district, there was not a strong local ECD system; the private and non-profit sector led the nurturing care collaboration. At the time, PATH was in the process of working with local partners at the national and subnational level to create policies, norms, and standards supporting the scaling of ECD programming. Strong leadership and support from goverment district.<br>An initial landsape report from 2012 found that there were few ECD programs or services beyond the health sector designed for children under 3.	While the government did not have a strong existing ECD programs, the government led this program by coordinating with the UN, and NGO partners. NGOs and intergovernmental orgs suported government agencies. Partners included: UNICEF, PATH, ICAP, GHI360/Covida, H2N. PATH employed a dedicated partnership coordinator to work with these partners. These partners were flexible with some leaving and coming through the course of the program.<br>Implementors worked with the Ministry of Health to revise the maternal and child data registers to better equip the government and other stakeholders to track the number of children receiving ECD services and those with suspected delays.	Government and external organizations	Good Health<br>Adequate nutrition<br>Opportunities for early learning<br>Responsive caregiving	The nurturing care collaboration implements responsive caregiving with health, nutrition, and early education sectors in the district. One example is how the NGO pathfinder integrates ECD into its family planning education by framing the education on contraception in the idea that it would allow parents more quality time with each child to nurture them. CCD is integrated into clinical work specifically into nutrition education.	Home and facility-based services (individual)	Not reported	Not reported	**Universal:** Delivered to children living in selected areas.	**Play:** Counselling on early learning based on CCD<br>**Print Materials:** Counseling cards, visual aids (i.e. poster)<br>**Other:** awareness messages broadcasted in radio during group sessions or home visits; district-wide mass media campaigns were disseminated	at least 438	Facility-based not reported; Community-based agent caseload ranged from 30 households to 10 households montly depending on the target population	Recruited and trained a preexisting workforce of CHWs and clinicians. Some are community-based service providers who reach children and caregivers at home or through structured play visits in a health facility.	Not reported	Content on responsive caregiving and early stimulation based on CCD was added in the latter half of the pilot to enhance providers' capacity to couseling caregivers.	PATH provided 4-5 day long training with classroom based and hands-on training adapted to different audiences (e.g. CHWs, clinicians). Now they use a train the trainers model with PATH training local program heads to train at the community level. PATH trains a cadre of government trainers and supervisors by incorporating ECD information into their standard trainings (i.e. WHO's Integrated Management of Childhood Illness (IMCI) package). These trained staff then build capacity with service providers, integrating content on nurturing care into their routine service delivery (including antenatal and postnatal care, child immunisations and growth monitoring, paediatric services, and community-based interventions). Content on responsive caregiving was added in the latter half of the pilot to enhance providers' capacity to support caregivers reponsiveness and positive interaction with children.	One-day refresher training four times a year	PATH staff serve as mentors.	Not reported	Not reported	Not reported	PATH supports structured mentoring wherein mentors observe staff counseling skills and provide feedback and coaching. Weekly review meetings, and monthly supervision meetings were strategies used with community-based implementation agents. Implementation agents who receive below 80% on the implementation checklist monitoring their performance receive hands on mentoring from supervisors.	Supervisors used an adapted WHO/UNICEF 24-item checklist to evaluate progress in interpersonal skills, nutrition, developmental screening, and counseling. The checklist identifies areas to be improved and how to tailor support. If a provider receives 80% on the checklist two times they no longer receive such hands on mentoring. They are only supervised and monitored quarterly. Supervisors were responsible for managing the reporting of developmental milestones and program activities which was critical to adapting the program. Collection and monitoring ECD indicator in the health information system.
**Rwanda** (low income)	Sugira Muryango	Rwanda's National Early Childhood Development Program collaborates with Vision 2020 Umerenge Program (VUP). VUP is a cash transfer program providing cash for work opportunities for Rwanda's poorest families along with health, education, development, and other financial services. ECD policy is also embedded in the national Economic Development and Poverty Reduction Strategies. Childhood daycare is available to children aged five years and older. Currently, Rwanda has the ECD priority of opening model ECD centers.	US based researchers collaborated with government ministry of gender and family promotion in partnership with the national commission for children, UNICEF, and the Imbuto Foundation. Additionally, program design and implementation was informed by community advisory boards.	External organizations and research project	Good Health<br>Adequate nutrition<br>Opportunities for early learning<br>Responsive caregiving<br>Security and safety	Modules of Sugira Muryango included topics of early stimulation and responsive caregiving, nutrition, hygiene, family conflict resolution, engagement of male and female caregivers. The original intervention was aimed to specifically meet the ECD needs of families affected by HIV/AIDS.	Home (individual)	60-90 minutes (time adjusted to fit families' needs)	Weekly, but designed to be adjusted to fit unique families' needs.	**Indicated:** Families were eligible if they have children aged 6-36 months, living in extreme poverty, families eligable for one of two versions of VUP (classic public works and expanded public works), families willing to participate. Families were excluded if there was a severe, active crisis in the family such as caregiver mental health issues or active separation.	**Play:** play, interactions with kids<br>**Other:** demonstration, try out, feedback to parents, and inclusion of fathers	4 (pilot trial); not reported (feasibility trial)	5 families per agent (calculated)	The pilot trial used bachelor-level educated implementation agents who were all fluent in English and Kinyarwanda delivered the intervention. The full trial trained implementation agents, called community based coaches, were selected based on the following criteria: lived in beneficiary households, aged 18 or older, can read, write, and count in Kinyarwanda, committed to young children, have time, recommended and approved by local community authorities at a village meeting led by the village chief.	Received $27 monthly to compensate for time spent: communicating with their supervisors, delivering CCD for 3 hours per day, and travelling to houses.	Intervention designers developed a comprehensive intervention manual.	During the pilot trial, coaches were trained for two weeks by the intervention developers and graduate level expert trainer with a background in ECD. For the full trial, trained supervisors delivered a three week training using role-play, active coaching practice, father engagement strategies, strategies on providing feedback, problem solving, referral and resource navigation.	Not reported	During the pilot trial, supervision was provided by the study investigators, a graduate level ECD expert, and a psychologist.<br>During the full trial, the bachelors level coaches served as supervisors. The supervisors were supported by trained psychologists who met with the supervisors twice a month.	No specific supervisor training reported. Supervisors in the full trial had previously been trained as implementation agents.	Not reported	Not reported	During the pilot trial, supervisors provided weekly phone supervision with implementation agents that consisted of reporting on activities, discussions as groups, and problem solving. A Rwandan program manager met with the "coaches" to review intervention delivery.<br>During the full trial, supervisors provided in-person supervision for three weeks after training and shadowed each CBC on one home visit. After that, they provided telephone supervision with peer support groups weekly. On a monthly basis, the supervisors facilitated a group discussion at the cell or sector office to address issues and problem solve as a group. During the third and sixth week of the intervention, supervisors shadowed the implementation agents and provided immediate feedback.	In the full trial, the home visits were audiotaped and the supervisors picked up the tapes fortnightly reviewed the recording to assess fidelity.
**Tanzania** (low income)	Integrated health, nutrition, responsive stimulation package	Antenatal and post-natal maternal depression prevalence are high in Tanzania; major risk factors linked maternal depressive symptoms were low socioeconomic status, food insecurity, lack of partner support, intimate violence, and living with HIV. Pareting and responsive stimulation have been identified as potential preventive approach to maternal mental health.	US based researchers collaborated with the Ifakara Health Institute in Tanzania.	Research Project	Good Health<br>Adequate nutrition<br>Opportunities for early learning<br>Responsive caregiving<br>Security and safety (CCT)	The Ifakara Health Institute HDSS implemented a home visit intervention that integrated responsive caregiving, health, nutrition, and conditional cash transfer components in the Kilombero and Ulanga districts. CCD is integrated into home visit to provide intervention-specific responsive stimulation component.	Home (individual)	30-60 minutes	Every 4-6 weeks	**Indicated:** Women/caregivers and their infants (if applicable) were eligible if (1) permanent resident of study village, (2) currently pregnant (self-reported) or have a child < 1 year of age, (3) and provide written consent for enrollment. Women/caregivers if they: (1) are currently enrolled in any other clinical trial or intervention study, or (2) do not provide informed consent.	**Play:** developmentally appropriate play and communication activities, toy making, and problem solving<br>**Other:** demonstration and feedback/coaching to parents	Not reported	Not reported	The project recruited female community health workers (CHWs) living in the study villages catchment area who had completed a one-year government certified CHW program prior to being selected for the intervention. The government CHW curriculum included two semesters that each covered seven topics	CHWs' salary was ~ 600,000 Tanzanian shillings (~$230) per month	Trained in a Tanzanian and Swahili adapted version of the UNICEF and WHO Care for Development package	CHWs received a one-week classroomintervention specific training that covered both theoretical and practical aspects of early childhood development, age-appropriate play and communication activities, counseling of caregivers, problem-solving, and making toys, and other materials.	A 3-day refresher training was conducted after 9 months of implementation.	One field coordinator supervised the CHWs throughout the intervention. Characteristics of the field coordinator were not reported.	Not reported	1	Not reported	Supervision included one-on-one biweely meeting with each CHW to report visit completion, a monthly meeting with all CHWs, monthly home visit spot-checks where field coordinator accompanied CHWs during home visits and provided feedback on intervention delivery.	CHWs level kept administrative records including the duration of home visits and the composition of messages delivered at each visit that were reviewed by the field coordinator once a month.
**Americas Region**																								
**Brazil** (middle income)	Criança Feliz	Two ECD-related milestones were the 1990 Child and Adolescent statute, and the 2016 Legal Framework for Early Childhood Development. The Criança Feliz program was finally implemented by means of a Presidential Decree, issued in October of 2016.	Social Assistance sector takes the leadership role in the multilevel implementation which is carried out through the existing National Unified Social Assistance System (SUAS).	Government funded (mainly federal funds to state (annualy) and municipalities(monthly))	Opportunities for early learning<br>Responsive caregiving	At each level of government (i.e. federal, state, and municipal), the program is expected to have an intersectoral committee which should foster collaboration between Social Assistance (lead sector) and Health, Nutrition, Sports, Human Rights, etc. for articulating the intersectoral actions	Home (individual)	45 minutes	one visit per month for pregnant women; weekly visits for children under 36 months old who are socially vulnerable or in at-risk situations; biweekly/monthly visits for children under 72 months old with disabilities	**Selective:** It started selective (just very vulnerable families enrolled in the conditional cash transfer program) and moved to include any family in social vulnerability that is registered in the National Database of Vulnerable Populations (CADÚNICO) is eligible.	**Print materials:** CCD checklist and forms in the home visit manual<br>**Play:** homemade toys including plastic water bottle with pebbles, and everyday household items like cups.<br>**Other:** demonstration, feedback to parents, and inclusion of fathers	20,000	~30 children or pregnant women per agent	Implementation agents must have at least a high school degree. Supervisors require college degrees.	Hire process happens at the municipal level and each municipality can establish specific rules and hiring process/contracts	An operational home-visiting manual (step-by-step home-visiting methodology based on the experience of Programa Primeira Infância Melhor (Better Early Childhood Program) and a protocol for training based on the evidence-based WHO/UNICEF CCD were developed in the early stages of scaling up.	The PCF training package includes 80 h of training, including 40 h in theWHO/UNICEF CCD methodology and 40 in the home-visiting methodology and it is operationalized through a multilevel training cascade strategy where master trainers from the federal and state levels support municipalities to train home visitors and their supervisors.	Short introductory/refresher virtual training on the CCD was cited as a strategy to increase training capacity, enhance quality, and reach more home visitors. In addtion, virtual training and continuing education process being developed to address highly sensitive topics to municipalities (eg. food insecurity, teen mothers).	**Municipal-level supervisor:** workers with advanced degrees meeting the governmental required minimum education of having an undergraduate diploma and professional registration in the following fields: psychology, social work, sociology, anthropology, domestic economics, occupational therapy, education, or music therapy.	Not reported	3,800	Up to 15	Municipal-level supervisors provide supportive supervision to home visitors. Municipal-level supervisors are supported by state-level staff providing technical assistance and mentorship.	Municipal-level supervisors are responsible for monitoring the quality of home visits. This data is used by municipality, state, and federal-level program leaders.
**Eastern Mediterranean Region**																								
**Pakistan** (low-middle income)	Pakistan Early Child Development Scale-up Study (PEDs)	The Lady Health Worker program started in 1994 to supplement the country's weak rural clinical infrastructure by providing care to families in rural, disadvantaged areas. In 2010, the Lady health Worker program employed 100,000 Lady Health Workers, delivering services to 60% of the population, mostly in rural areas, using community women to implement a broad range of family health intiatives. In 2007, health governance shifted from federal to provincial and there was a need for provincial health leadership to strengthen capacity.	UK, USA, and Pakinstani investigators based at Aga Khan University partnered with national and local lady health worker program leaders	Research project and external organisations	Good Health<br>Adequate nutrition<br>Responsive caregiving	CCD is delivered to the responsive caregiving and combo treatment arms, the nutrition groups received nutrition education and multiple micronutrient powder for children older than 6 months. All treatment arms inluded the basic health advice that Lady Health Workers already deliver.	Home (individual) and out of home (group)	Individual 30 minutes; group 80 minutes	Monthly for group and home visits	**Universal:** every child under the age of 2 in the catchement area of the clusters receiving the intervention is eligible, enrolled infants can be 0-5 months.	**Print Materials:** printed job aids and picture book given to each family<br>**Play:** resource kits including play materials and homemade toys including plastic water bottle with pebbles, and everyday household items like cups.<br>**Other:** Lady Health Workers also brought tea or juice and biscuits during group sessions	40	5-7 households per day per agent	Used existing Lady Health Worker workforce to deliver intervention. Lady Health Workers have the following selection criteria: 18-45 years old, live locally to the village, married preferably, at least eight years of education, accepted by community. Each Lady Health Worker oversees a catchement area of 100-200 households, some of which have young children eligible for CCD.	Lady Health Workers received monthly salary equivalent to $85 USD		ECD facilitators provided training to Lady Health Supervisors and Lady Health Workers. Management team (including ECD facilitators) were trained for five days. Lady Health Workers were trained 20 Lady Health Workers at a time. The responsive caregiving training lasted 3 days plus an additional 2 days for nutrition training for those two treatment arms. Prior to the interventin, all Lady health workers received fifteen months of training in two phases. Phase 1 is five days a week for three months classroom topical learning covering: immunizations, diarrhea control, maternal child health, family planning, nutrition, and hygiene as well as skills of community organization and interpersonal communication. Phase 2 is twelve months working in the field for 3 weeks as a time with one week per month of classroom training.	One day per month all Lady Health Workers receive refresher training on specific topics with an opportunity to discuss work challenges. The additional responsive caregiving and nutritoin training for PEDS used 2-5 days of this refresher training. During the implementation, every 6 months atht elocal health office there was a one day refresher training designed by supervisors identifying skill gaps of Lady Health Workers.	**Lady Health Supervisors:** Lady health supervisors are existing supervisors that were trained to supervise CCD delivery in additional to the base workload. Supervisors had access to a vehicle and earned ~$77 monthly.<br>**ECD Supervisors:** An additional cohort of supervisors was trained to proviede extra support by to the lady health supervisors.	3 months training	6	20-25	Supervisors helped implementation agents problem solve and provided advice after monitoring home visits and group sessions.	The supervisors would contact the implementation agents at least twice a month with at least one contact being a site visit where she would conduct a supervisory checklist.
	Sustainable Program Incorporating Nutrition and Games (SPRING)	Rural health services are delivered by the Lady Health Worker Program.	Researchers based in India, Pakistan, and the UK worked with the Lady Health Worker Program.	Research project and external organisations	Good Health<br>Adequate nutrition<br>Responsive caregiving	This intervention evaluates the integration of a maternal psychosocial wellness intervention with both the baseline Lady Health Worker program that addresses child health and nutrition and the enhanced SPRING program that focuses on responsive caregiving and nutrition.	Home (individual)	Not reported	Monthly	**Universal:** Pregnant women (5-7th month), immediately post birth, post partum period, infancy to 2 years. Delivered to all mothers in the clusters assigned the intervention.	Not reported	13	Not reported	Used existing workforce of lady health workers.	Not reported	Not reported	The Lady Health Worker of this enhanced program were trained for 5 days of classroom training and 1 day of field training. The maternal psychosocial wellbeing component was trained on in the first two days of the five day training. Training used short video clips, role plays, lectures, discussions.	Second training lasted three days.	The supervision of the maternal psycho-social program was integrated into normal supervision activities.	Lady health supervisors were trained to facilitate supervision.	Not reported	Supervisors met with a group of 10-15 Lady Health Workers every month for 2 hours.	Solutions were generated by group brainstorming, not just individual supervisor.	Not reported?
**Europe Region**																								
**Turkey** (upper-middle income)	Care for Development	There is a weak health system with an absence of well-child visits	Intervention led by a team of pediatricians based at the Ankara University School of Medicine.	External organization	Good Health<br>Responsive caregiving	Delivered CDI responsive caregiving training at the end of pediatric visits.	Outside of home (individual)	10 minutes	Single-visit, intervention not repeated	**Indicated:** children under 24 months who attended the outpatient clinic with minor or no illnesses	**Play:** Physicians showed mothers examples of age-appropriate play with example homemade toys to enhance communication and ECD stimulation	1	~60 children per agent	Pediatricians with similar background and experience at the outpatient clinic	No additional compensation in addition to pediatrician salary	Not reported	Physicians trained on CDI, details Not reported	Not reported	Researchers supervised visits and used the Physician Counsling Skills Scale to evaluate the physician's competence of counseling caregivers.	Not applicable	Not applicable; no new categories of workers were created to specifically deliver CCD. Pre-existing providers were trained to deliver CCD to families so the CCD	Not applicable	Not applicable	Clinic nurses conducted weekly home visits during the child's first month or two after birth and monthly up to age 2 years, to monitor the infant's health and advise families on child nutrition and preventive care.
**Kazakhstan** (low-middle income)<br>**Tajikistan** (low income)<br>**Kyrgyz Republic** (low income)	Better Parenting Initiative (Kazakhstan), IMCI with CCD (Tajikistan, Kyrgyz Republic)	Kazakhstan: The country has a policy environemtn supportive of ECD. There is strong government regulations on ECD in health centers. CCD became embedded in the Better Parenting Initiative within the Ministry of Health. Health centers all have Healthy Baby rooms.<br>Tajikistan: There is a Child and Adolescent Health Policy that promotes psychosocial stimulation and development, but and it is not well incorporated into the health system as yet and there is no dedicated ECD framework. Medical schools integrate ECD training.<br>Kyrgyz Republic: There is no ECD policy at the time of implementation. There are policies for health sector reform but they do not include strong ECD components. Health centers all have Healthy Baby rooms. IMCI is integrated into physician training at medical schools consisting of 16 hours of development, but not on child development.	Kazakhstan: External ECD experts collaborating with UNICEF and the local health system<br>Tajikistan: Program was initially initiated with support from trained experts from Kazakhstan. The program is driven by an adviser to the president who is a strong advocate of ECD. ECD experts from neighboring countries and internationally collaborate with medical universities and the health system. The Aga Khan foundation halso trains community volunteers.<br>Kyrgyz Republic: UNICEF was involved in the initiation but the program is mostly advised and suported by the WHO. International ECD experts conducted the initial training.	Kazakhstan: External organization then taken over by government<br>Tajikistan: External government<br>Kyrgyz Republic: External organizations with contributions from government	Good Health<br>Adequate nutrition<br>Responsive caregiving	CCD training was first provided to health care workers who integrate CCD activities with ongoing health and nutrition services at health services.	Home (individual) and outside the home (individual)	Not reported	CCD provided when children come to health centers for other reasons, weekly home visits for infants aged 0-2 months	**Universal**: CCD delivered to all infants who attend health center visits	**Print Materials:** Mothers Card (all), IMCI Booklet (all), education modules (Kazakhstan), child development diaries (Kazakhstan), child care leaflets (Kazakhstan and Tajikistan)	Kazakhstan: not reported<br>Tajikistan: 599<br>Kyrgyz Republic: not reported	Not Reported	Kazakhstan: Nurses were the first cohort to be trained in CCD. All public healthcare workers are eligible to be trained. Medical students are trained in ECD.<br>Tajikistan: Community health volunteers were trained first. In certain settings, all health workers were trained.<br>Kyrgyz Republic: Initially trained only family doctors, pediatricians, and physician assistants. Some nurses were trained later.	Does not report any additional compensation in addition to typical compensation as a public healthcare worker.	Adapted UNICEF materials	Kazakhstan: Initial training by ECD expert Dr. Jane Lucas. Basic training 5 days for nurses and medical assistants. WHO trained national IMCI trainers from medical school staff.<br>Tajikistan: Initial training delivered by staff from Kazakhstan. CCD is only part of regular IMCI training when a donor funds for it. CCD training is 3 days of the 9 day IMCI training. Medical schools integrate ECD training.<br>Kyrgyz Republic: Initial training by ECD expert Dr. Jane Lucas. Basic training 5 days for nurses and medical assistants. WHO trained national IMCI trainers from medical school staff. CCD now a part of all IMCI training since 2006 only for phsycians and physicians assistants. The entire IMCI training lasts 12 days including CCD. Very few nurses are trained in CCD. IMICI training for nurses is a 6 day program.	Kazakhstan: All staff trained in IMCI are trained in CCD every 3 years.<br>Tajikistan: Not reported<br>Kyrgyz Republic: Health workers retrained every 5 years.	No formal supervision or mentorship reported. Indicators on caregiving education is added to the IMCI form.	Not applicable	Not applicable; no new categories of workers were created to specifically deliver CCD. Pre-existing providers were trained to deliver CCD to families so the CCD approach was fully integrated into existing services.	Not applicable	Not applicable	Clinic nurses conducted weekly home visits during the child's first month or two after birth and monthly up to age 2 years, to monitor the infant's health and advise families on child nutrition and preventive care.
**South-East Asia Region**																								
**India** (middle income)	Project Grow Smart	India's integrated child development service (ICDS) is the largest ECD program. Launched in 1975 the government funded Integrated Child Development Services (ICDS) provides nutrition and health services to all pregnant and lactating women and their children. There are 1.4 million Anganwadi workers who deliver services to children and the program is led by the Ministry of Women and Child Development. Village-based Anganwadi centers provide preschool education and early child nutrition interventions. Local communities access health services and education via the Anganwadi and Accredited Social Health Activist (ASHA) programs. The National Rural Health Mission also implements ECD nutrition interventions.	The National Institute or Nutrition based in Hyperabad, India coordinated a team of partners from three countries (India, US, Canada), four institutions (NIN, University of Maryland, Johns Hopkins Bloomberg School of Public Health, Micronutrient Initative), and seven disciplines (nutriton, developmental psychology, public health, pediatrics, biostatistics, epidemiology, and biochemistry). Local village leaders, the Indian Council for Medical Research, and the Indian Health Ministry's Screening Committee were consulted and approved the project.	Research project	Adequate nutrition<br>Responsive caregiving	The RCT tested responsive caregiving and micronutrient supplementation arms separately as well as a combination arm. CCD was implemented in the responsive caregiving group and nutrition education and micronutrient supplementation was provided in the nutrition arms.	Home (individual)	35 minutes	Fortnightly	**Indicated**: Children aged 6-12 months were examined by the project physican who determined if they should be included if they did not have severe developmental delays, congenital illnesses, or severe anemia.	**Print materials**: books<br>**Play**: locally available toys and play materials including rattle, small steel tumbler, small steel bowl and spoon, basket and small blocks, slate and slate pencil, ball, doll, and small towel<br>**Other:** portable, laminated flipchart	36	~20 families per agent	Implementation agents were newly trained village women who had at least a primary education and were recommended by Anganwadi workers or village leaders.	Village-level workers receive 1,000 rupees (~$18.40 USD) monthly	Supervisors are trained using operation manuals.	The lead interventionist is responsible for training, supervising, and managing the intervention team.<br>Home visitors are trained intensely for 2 days at the National Institute of Nutrition with the project staff, then are trained in the villages for an unreported amoung of time.<br>Supervisors train the home visitors on study objectives, protocols, general intervention strategies of personalizing home visits and engaging caregivers, using study materials, and using the support of the supervisors.<br>Training involves home visit demonstrations, role playing with practice sessions.	There is one day of training every 3 months and weekly review sessions where the supervisors continue to practice with roleplaying and are evaluated with quisses. During weekly review sessions supervisors provide supportive feedback.	Four village level supervisors are recruited from graduates with backgrounds in either home secience or child development. They recruit and enroll the participating families and monitor implementation. They meet every other week with each home visitor to review progress and restock intervention materials. Supervisors meet with each other and the group implementer monthly to review records and discuss their experiences.	Supervisors are trained over one month on operating procedures, completing monitoring forms, and general support techniques. They are provided with training and operation manuals, which are prepared by the investigators (and include job descriptions and standard operating procedures, including instructions on the completion of monitoring forms and the FAQ). The training is completed over 1 month and includes practice role-plays and completion of monitoring forms.	4 village-level worker supervisors, 36 village-level workers.	Each village-level worker has a caseload of approximately 20 participants.	Supervisors meet with individual village-level workers every other week to help problem solve, replenish product supplies, review participant progress, and to discuss problems/solutions in increase program success related to schedulling and intervention visits.<br>Supervisors complete an Observation Record checklist following the monthly home visit observations; the record is to assesses the quality of the interactions between the village-level worker and the mother. They review these checklists during supervision to improve intervention quality.	Supervisors accompany home visitors on monthly visits where the complete an observation record to monitor the quality.<br>In the infant phase, the village-level worker supervisors recruit and enroll the participants and supervise and monitor the implementation of the intervention.<br>The Home-Visit Evaluation Form is completed by the village-level worker after every contact to address participant compliance and questions/concerns, and note village-level worker advice/encouragement.<br>Home-Visit Evaluation Forms are reviewed by supervisors weekly to verify fidelity and to address any concerns raised by mothers or the village-level worker.
	Sustainable Program Incorporating Nutrition and Games (SPRING)		Researchers based in India, Pakistan, and the UK worked with Anganwadi and Accredited Social Health Activist (ASHA) workers	Research project and external organization	Good Health<br>Adequate nutrition<br>Responsive caregiving	This intervention evaluates the integration of a maternal psychosocial wellness intervention with both the baseline Lady Health Worker program that addresses child health and nutrition and the enhanced SPRING program that focuses on responsive caregiving and nutrition.	Home (individual)	Not reported	Monthly	**Universal:** pregnant women (5-7th month), immediately post birth, post partum period, infancy to 2 years. Delivered to all mothers in the intervention areas.	**Print materials:** Adapted CCD content and other nutrition and health content into a Kilkaari Intervention Booklet and picture cards to guide home visits	Not reported	Not reported	Trained a new cadre of workers recruited from the community	Not reported	Not reported	First training is five days in length	second training is three days long.	Not reported	Not reported	Not reported	Not reported	Supervisors are empathetic to workers' issues, help problem solve, and support with new strategies. Individual supervision consists of giving individual feedback, and occurs in the field. The group supervision addresses common issues and challenges, provides peer group learning, and is conducted in the health center.	Research assistants collected data on child development and adversity indicators at 12 and 18 months of age. Individual supervision visits involved measuring quality and performance,
**Western Pacific Region**																								
**Vietnam** (low-middle income)	Learning Clubs	Existing maternal care services do not include maternal mental health services.	Pilot program conducted in collaboration between the Research and Training Centre for Community Development, the Ha Nam Women's Union, Jean Hailes Research Unit at Monay University in Australia, University of Melbourne Department of Medicine.	Research project, external organizations, and local organizations	Good Health<br>Adequate nutrition<br>Responsive caregiving	CCD responsive caregiving support was integrated with the Thinking Healthy program to improve maternal mental health. The intervention used exisiting commune health system as a base for intervention activities used exisiting commune health system as a base for intervention activities used existing staff at the Women's Union. The intervention itself included modules on pregancy health, support of mothers, maternal mental health, infant development, and responsive caregiving. The intervention itself combines CCD with the Thinking Healthy Program, WHO guides on newborn care, Metanl health GAP, IMCI, and the national feeding program.	Home (individual) and outside of home (group)	Not reported	Not reported	**Universal:** universally delivered to children and caregivers living in selected rural area	**Print materials:** printed leaflets<br>**Play:** dolls<br>**Other:** DVD, laptop, DVD player, projector	21	~19 caregivers per agent (calculated)	Community-based lady social workers from the Women's Union and community-based doctors and nurses at the commune health clinic deliver the intervention.	Not reported	Not reported	Training includes six courses.	Not reported	9 district and provincial level Women's Union staff trained as supportive supervisors.<br>Included in the list of prorgam-related costs were: training and transportation operation, facilitator manuals, training courses for provincial trainers, and training courses for commune facilitators.	Not reported	Not reported; however, supervision trips were included in the list of program-related costs	Not reported	Not reported; however, supervision trips were included in the list of program-related costs.	Questionnaires were used to assess mothers' quality of life, prevalence of common mental disorders, productivity. Outcomes of faimly environment were also measured with various adapted scales and questionnaires.
**China** (low-middle, upper-middle income)	IMCI with CCD	The Integrated Management of Childhood Illness (IMCI) expanded within China's health sysem in 2001. The Care for Development (WHO) guidelines were adapted to China and first introduced through a national pilot course in Shanghai in December 2002.	Not reported	External organization	Responsive caregiving<br>Opportunities for early learning	Not reported	location not reported (individual)	30-60 minutes	2 sessions over 6 months	**Universal:** Delivered to all children in the selected villages.	**Print materials:** 'Mother's Cards' were adapted for China from the WHO global CFD materials. The card showed age-specific messages for promoting effective play and communication between caregiver and child. The pictures use clear line drawings, simple language.	Not reported	Not reported	Not reported	Not reported	Not reported	All members of the research team receieved a pilot training course in CFD and received WHO technical support as part of China's national IMCI activities.	Not reported	Not reported	Not reported	Not reported	Not reported	Not reported	All children (in both control and intervention group) received a developmental assessment at baseline and after 6 months. A questionnaire was administered to all families on child development and child rearing at baseline and after 6 months. Families in the intervention group received counseling using the Care for Development Mother's Card and were provided with a copy of the Mother's Card.<br>The questionnaire (27 questions total) was administered twice (once at baseline and once after intervention was completed) by interview to all mothers at the same time as the developmental assessment. Questionnaires were administered by health professionals trained as part of the research team.
	Integrated Early Childhood Development Programme (IECD)	The Wolrd Health Organisation and United Nations International Children's Emergecy Fund Care for Child Developmet package was used as a counselling tool to help families build stronger relationships with their children.	The county was in charge of managing, coordinating, and supporting community leaders and workers.	Research project	Good Health<br>Adequate nutrition<br>Opportunities for early learning<br>Responsive caregiving<br>Security and safety	This study was possible through the involvement of workers from various sectors including village doctors, volunteers, early-stimulation professionals, maternal and child health professionals and social workers; also the mobile resource unit allowed for as a critical "multisectoral working mechanism" for quality assurance and transportation of workers to rural areas.	outside of home (individual and group)	Clinic visits not specified; ECD center sessions were provided by age-group for child-caregiver activities 3-4 times a week	Bimontly visits from the Health Bureau and Women's Federation and Civil Affairs Bureau	**Universal:** Delivered to all children in the selected counties.	**Play:** Storybooks, toys, touchscreens were all used to help accomodate play activities for children and their caregivers in the groups.	Not reported	Not reported	The full-time volunteer who was recruited to run activities at the ECD center had to have completed 8 years of schooling, have parenting experience, and be familiar with (if not from) the village.	Not reported	Not reported	Training for village program staff was provided by and based in the village ECD center.	Not reported	Not reported	Not reported	Not reported	Not reported	A multisectoral working group usually headed by county (vice) governor was set up at the county-level to manage, coordinate, and support all the community leaders and workers involved in the intervention.	Investigators administered face-to-face interviews with caregivers, through a structured questionnaire to collect data on demographic characteristics of the children, caregivers and their families, and other factors that would potentially impede or facilitate ECD.
