# Supplementary material for: Pathways to scale up early childhood programs: A scoping review of Reach Up and Care for Child Development
Source: PLOS Glob Public Health. 2023 Aug 9;3(8):e0001542. doi: 10.1371/journal.pgph.0001542 (PMC10411826; doi:10.1371/journal.pgph.0001542)
Supplement: S7 Table — (PDF) [file pgph.0001542.s009.pdf]

S5b Table. Implementation Outcomes of Reach Up programs

Country	Program Name	Appropriateness	Feasibility	Acceptability	Adoption	Fidelity	Adaptation	Penetration	Sustainability	Implementation Cost	Scaling	Program Outcomes	Impact
**African Region**													
**Zimbabwe** (low income)	Modified Reach Up	Intervention: Curriculum and manuals were found to be appropriate by caregivers. It was important that the mothers were able to incorporate the learnings into their daily lives. Toys were not durable enough.<br><br>Workforce: HVs wanted additional training to address different home environments, and supervisors wanted more guidance.<br><br>Training: Curiculum and manuals were found to be appropriate by home visitors.	Intervention: Mother-home visitor relationships and community toy production were key to program feasibility. Program was adapted from weekly to fortnightly to improve feasibility for program staff.<br>Training: Strong training of home visitors was key to feasibility. Visits were most successful and feasible for HVs when they prepared prior to each visit.<br>Monitoring: Close contact with supervisors supported HV activity.	Intervention: Mothers reported enjoying the program and thought it should continue. . Caregivers were initially hesitant about toy material but found them acceptable after gaining familiarity and being guided by HVs.. Caregivers valued that toys were made from local, recycled materials; however, toys were not durable enough for caregivers. A few mothers did not accept toys and that was a barrier to their engagement. Positive relationships with HVs were also valued by caregivers	Intervention: The relationships between HVs and mothers and children was a main factor contributing to mother's ability to complete the activities.	Training/Monitoring: HV preparation before each visit improved visit success. Close contact with supervisors to trouble shoot problems also helped ensure success. The observation checklist was used to determine visits were conducted well over 90% of the time.<br><br>Intervention:The interactions between caregiver and visitor and child were warm. This evaluation revealed that home visitors could improve encouragement of mothers and the inclusion of mothers' opinions.	Intervention: Pictures were revised to be reflective of local culture.<br>Intersectoriality: Program adapted to be scaffolded onto the existing ECD program with ECD centers.	Not reported	Not reported	Not reported	Not reported	Not reported	Not reported
**Madagascar** (low income)	Early Stimulation	Intervention: Challenges included insufficient time for home visits and parents' lack of interest in topics.	Intervention: Play materials were not able to be kept in the house due to cost constraints, which may have been a challenge to sustaining parent engagement in activities and therefore also to improving childrens' ECD outcomes.<br>Monitoring: It was not feasible for NGO supervisors to visit implementation agents frequently, perhaps contributing to the small program effect size.	Not reported	Intervention: Parents' lack of engagement with community health workers was a challenge.	Not reported	Not reported	Not reported	Not reported	Not reported	Not reported	Not reported	The program did not include child interactions with other caregivers, which perhaps limited the effect.
**Americas Region**													
**Jamaica** (low income)	Jamaican Home Visiting Program	Intervention: Books, containing all or mostly all pictures, were developed to be culturally appropriate, reflecting people and environments familiar to the families.	Intervention: Home visiting was chosen for optimal feasibility, it was lower cost and more available than centre-based interventions, and the most disadvantaged women often did not or could not come to centres. Reducing visit frequency from weekly to fortnightly was found more feasible.	Not reported	Not reported	Monitoring: Fidelity and implementation quality of the CHW activities was measured by supervisors (research team staff) every 6 weeks. The median rating was 8/9 suggesting satisfactory fidelity.	Intervention: Program curricula were adapted to include local songs and games	Not reported	Training: There is a need to build the implementation team's training capacity.	Intersectoriality: If health and nutrition staff could include child development activities in their services, additional benefits for children could be acheived for lower cost than stand-alone services.	Monitoring: Future supervision should be carried out by local staff, and not a research team, to assist in scaling up.	Intervention: Initial feasibility and effectiveness testing showed significant improvements in development for children, and improvements in mothers' knowledge and practices of childrearing. Four years after the end of the intervention, children who received any of the interventions had higher scores than control subjects on numerous tests measuring cognitive function and educational attainment. There were no sustained benefits of the intervention on cognitive function and educational attainment shown in late adolescence, but 20-year follow-up showed the intervention compensated for early developmental delays and reduced later-life inequality.	From the JHV, this methodology has shown that benefits to education, mental health, income, and reduction in violent behavior result from the program.
**Colombia** (middle income)	Home-based Early Childhood Development Intervention	Intervention: Mothers who were able to see a benefit to their child's development were more confident about using and making the play materials. Mothers mentioned wanting more durable toys, and thought a toy-making workshop should be part of future interventions.	Intervention/monitoring/program emergence: A challenge to feasibility was the rural location and difficult transportation for supervisors to travel to home visiting sites.<br><br>Traininng: established, standardized framework for curricula content would improve feasibility and ongoing HV and supervisor support.<br><br>Workforce: Feasibility dependent on strong recruitment strategy.	Not reported	Workforce/monitoring: Challenge of overburdened supervisors could be addressed through hiring of local supervisors (if necessary with lower qualifications) to limit travel time and increase frequency of supervisions for HVs (which improves skills, motivation, and commitment to program). Further suggestion that, where possible, it may be helpful to increase the HVs' required level of skills for employment.	Monitoring: Supervisors distributed reminder bulletins, and sent reinforcement messages, and visited HV's communities every 7-10 weeks to monitor fidelity.<br>Training/Workforce: Need for increased coaching, training, and technical assistance; hiring of local supervisors to limit travel time and increase supervision opportunities for HVs.	Intervention: Materials were left at the home for the week after the visit and changed out weekly.<br>Supervision: Meetings were held every 9 weeks given the large catchment areas and subsequent distances between HVs and supervisors.	Not reported	Workforce: Hiring full-time home visitors could improve commitment to the program and decrease work for supervisors.<br>Intersectoriality: Intervention would be more sustainable if program were integrated into governmental policy, allowing for more localized visits, more frequent HV contact with families, and reduced HV turnover.	Intervention: Approx. USD $500 per child per year (about 1/3 the cost of the national center-based programs). Cost ratio benefit for home visits calculated at 2.6.	Program emergence: Use of existing health and program infrastructure allowed for replicability and feasability at scale.	Significant improvements were found at the 18-month follow-up, but no statistically significant gains in ECD were found at the second follow-up. Outcomes are thought to be more successful overtime with an ongoing intervention. Micronutrients were not found to have a positive impact on childrens' growth or development.	Even small to moderate improvements in cognitive development are thought to be sustained and are important to addressing and decreasing the gap between disparities in ECD outcomes in Colombia.
	Enhanced FAMI	Intervention/training/intersectoral: program was considered appropriate due to the curricula (providers); the use of toys and materials (caregivers); and the collaborative approach (providers).	Intervention/training/intersectoral: Program was considered feasible to implement due to the curricula (providers); the use of toys and materials (caregivers); and the collaborative approach (providers).	Intervention: Caregiver appreciation of toys and materials. Home visits were perceived as more personalized services, while group sessions were considered more fun, and sharing and learning from a peer were appreciated by mothers. No advantages were reported for home visits and participants liked the group and home visits equally.	Intervention: The sessions were poorly attended initially until the team involved families and communities in intervention adaptation and implementation.<br>Program emergence: Incentivization strategies were considered and attempted, but not selected as the team was mindful of future limitations of incentivations while scaling up. Instead, the researchers focused on communication, engagement, and awareness.	Not reported	Supervision: Meetings were held every 9 weeks given the large catchment areas and subsequent distances between HVs and supervisors. Mentors distributed one page bulletins regarding best-practices to HVs and sent short texts to HVs every month to reinforce key advice. HVs were also encouraged to call mentors for advice as necessary.	Not reported	Program emergence, training, monitoring: Improved through use of existing government infrastructure, ability to leverage explicit country commitment, and transferring of supervision and training responsibilities to government workers.	Intervention: Calculated at USD $320 per child per year (compared to the center-based care estimated at US $1100 per child per year).	Program emergence, training, monitoring: Improved through use of existing government infrastructure, ability to leverage explicit country commitment, and transferring of supervision and training responsibilities to government workers.	A positive impact on ECD outcomes, reduced levels of stunting, improved parental practices, improved caregiving environment. There were no harms reported by any participants.	Not reported
**Brazil** (middle income)	Responsive Caregiving and Early Learning Program	Intervention: Activities were most successful when mothers could easily integrate them into day to day activities. Fewer home visits could feasibly still have a positive impact on ECD outcomes.<br><br>Workforce: The human resources available to support the workforce, case load, scheduling, training and supervision all are important to ensuring feasibility.<br><br>Traininig: HVs indicated expanded training and material on community problems such as household violence would be more appropriate for household settings.	Intervention: Activities were most successful when mothers could easily integrate them into day to day activities. 73% of caregivers reported being able to incorporate new activities into daily life. Fewer home visits could feasibly still have a positive impact on ECD outcomes. A challenge to feasibility was finding local materials to make and replace toys.<br><br>Workforce/training/monitoring: The human resources available to support the workforce, case load, scheduling, training and supervision all are important to ensuring feasibility.	Intervention: Mothers who were able to see a benefit to their child's development were more confident about using and making the play materials. Mothers mentioned wanting more durable toys, and thought a toy-making workshop should be part of future interventions. Caregivers were initially hesitant about toy material but found them acceptable after gaining familiarity and being guided by HVs. Caregivers valued that toys were made from local, recycled materials; however, toys were not durable enough for caregivers. A few mothers did not accept toys and that was a barrier to their engagement.<br><br>Program emergence: Acceptability is strengthened when program designers and adaptors have a strong strategy to understand local design factors that should be incorporated into curriculum content and program delivery.	Intervention: Visits were reported as happy.	Monitoring: The observation checklist was used to evaluate home visit quality. The quality of CDA visits were conducted well 90% of the time.High quality, consistent supervision is essential to program fidelity and quality implementation.<br><br>Intervention: Interactions between visitors and children were warm, understanding, and cooperative.	Intervention: After input from parents, toys were adapted by program implementors to be more attractive in color and texture, while still maintaining key attributes to optimize ECD learning. For example, bottles caps were kept in primary colors (as opposed to painted with designs) to be used for color identification purposes.<br>Training: Community health assistant trainings were completed in 2 segments to accomodate workers' schedules.	Not reported	Intervention:Need for increased guidance for supervisors and HVs, with an emphasis on role plays in trainings.<br>Training: Additional trainings would ensure general confidence and preparedness for different home environments.	Not reported	Workforce: Retention of workers was a consistent challenge.	Not reported	Not reported
**Peru** (middle income)	Cuna Más	Intervention: A co-management model allows the community to give direct input into the operations and resource administration of the program.<br><br>Training: A cascade model of training allows for input from the various geographic and personel levels of the intervention.	Training/Monitoring: Staff across all levels emphasized the importance of training and continued support and supervision.<br><br>Workforce: Overlapping and poorly defined roles/responsibilities at the local level were a burden for community workers.<br><br>Program emergence:Transportation cost burden of getting to remote communities is a burden.	Workforce: Program staff were more motivated when they could see the direct value and impact of their work on the children and families, despite challenging work conditions and low salaries.	Workforce/training: Challenge to recruit and retain HVs given the longest possible contract for staff was 6 months; estimated 33% turnover in 2016. Request for additional training to clarify workforce roles and responsibilities.	Workforce: Local service delivery was inconsistent due to overlapping and confusing roles.	Not reported	Program emergence/scaling: 85,000 families received Cuna Más services, or approximately 32% of the target population. Cuna Más operated in about 580 out of 713 eligible rural districts.	Workforce: Recruitment and retainment of HVs. A more robust and diverse cohort of HVs could be reached by relaxing the requirements for program workers. Opportunities for career advancement, professional development, longer job contracts, and more balanced workloads would help minimize workforce turnover.<br><br>Intervention: Interventions were run through clinics using existing staff.	Intervention: Budget is controlled by the Ministry of Economy and Finance and was financed using a results-based budgeting approach. The total cost of the home visiting program in 2016 was approx. USD $480 per family (1525 soles).<br><br>Workforce, Training:The largest cost drivers were the salaries and stipends (~60%), central program management costs (16%), materials and resources for community actors (13%), and training and support costs and regional office overhead costs (~11%).	Program emergence: Situational analyses identified the existing public infrastructure and the government commitment.Researchers built on long-standing relationships with local partners and in particular the government health sector.<br><br>Intervention: Interventions were run through clinics using existing staff.	Positive impacts on parenting practices and ECD outcomes, and decreased harsh childhood discipline.	Positive impact on child development, especially cognition and language Follow-up evaluations showed no positive impact was sustained after 2 years.
**Eastern Mediterranean**													
**Syria, Lebanon, Jordan** (low income)	Modified Reach Up and Learn	Training: Expressed need for new training modalities that would suit community context (e.g. part-online modules to reduce transportation need).	Workforce: Some home visitors reported long walks/difficulty with transportation and weather, and concerns about safety (especially when conducting visits alone).<br><br>Training: Expressed need for new training modalities that would suit community context (e.g. part-online modules to reduce transportation need).<br><br>Program emergence: long-distances and transportation.	Workforce/training: HVs liked that the curriculum included a full day of toy making built into their monthly visit schedule. Communicate and adapt scheduling expectations to reflect personal/cultural time preferences of HVs. Caseloads should be low (3-4/day) in order to promote retention and avoid burnout and fatigue of home visitors. HVs expressed interest in receiving continued or futher training on relevant topics.<br>Intervention: HVs noted that families were more likely to understand/adopt the program when the activity benefits/importance were explained ahead of time, when there was evidence of their child's learning new skills, and when activities were modeled using household items/chores/everyday activities as opportunities for ECD learning.	Workforce: High home visitor turn over could be addressed through regular trainings and dedicated staff for capacity-building.	Not reported	Intervention/training: Adaptation and language translation was key to capturing cultural appropriateness and meaningfulness of curriculum. The training manual was adpated to include information on families with mutliple children present, caregiver well-being and psychosocial support, child protection and safeguarding, and families with children with disabilities. Storybooks, pictures, and songs were adapted to be flexible, community-specific, and relevant.Toy making guidelines helped identified local/available materials, toy safety criteria, and lessons for what to do when there were changes in the distribution of materials that were typically recycled to make toys. Curriculum was professionally translated into Modern Standard Arabic and then reviewed by the Arabic Resource Collective for technical accuracy. Further changes were made in each country to reflect local relevance and comprehension.	Intervention: Challenge to enroll new families and explain benefits of the program; the idea of HVs focusing on play and communication for young children was novel.	Workforce, Training, Supervision: Set clear expectations about HV conditions and commitments; set minimal requirements for education and previous work experience for HVs; offer in-service professional development to address job challenges; provide regular supervision and one-on-one coaching; employ country-specific approaches to HV retention.<br><br>Intervention: Provide up-to-date referral information for families, continue curriculum revision for cultural relevance and context, improved data collection approaches, pilot new training modalities that reflect needs and capacities of the community (partially online, digital, etc.), and maintaining low costs through use of para-professionals.	Intervention: HV stipends were the largest cross driver across all contexts, at typically USD $250-400 per month, and based on the number of days worked and local labor laws. The stipend impact on cost per beneficiary was influenced by caseload size (number of visits, landscape and travel, country-specific staffing decisions) and cost-sharing with other programs.<br><br>Workforce: Combining program delivery with local community health workers reduced visit times and notably reduced stipend costs. While costs for materials and toys could be high, they were ultimately less significant cost drivers than stipends.	Program emergence: Need to coordinate with governments to continue scaling.	Caregiver behavior changed to result in increased emotional engagement in activities to promote ECD, including loving and playful interactions.	Not reported
**South-East Asia Region**													
**Bangladesh** (low income)	Integrated psychosocial stimulation and unconditional cash transfer	Not reported	Workforce: Community health workers were locally recruited with the help of community leaders considering that they should have easy access to the participants' houses and villages.	Not reported	Program emergence: Highly motivated experienced child development research group	monitoring: female supervisor with master's in psychology supervised to ensure quality of the intervention. Daily check sessions conduction form by a male field supervisor.	Intervention: Reach Up curriculum including nutritional and personal hygiene messages for mothers and children translated to Blanga language.	Not reported	Not reported	Not reported	Not reported	Children receving the psychological stimulation and unconditional cash transfer had significant improviment in their cognitive, language, and composite scores compared to children receiving only unconditional cash transfer. The effect was larger when compared to the comparison group (not receiving unconditional cash transfer). Similar findings were found regarding maternal self-esteem and home stimulation. No effect on children growth and mothers BMI were observed.	Not reported
	Integrated responsive stimulation, maternal mental health, nutrition, WASH and lead exposure prevention interventions (RINEW)	Program emergence: The integrated curricullum was developed based on the assessmemnt of gaps on knowledge about pregnancy, and factors affecting early childhood development were reported as needed.<br><br>Intervention: appropriateness of duration of sessions, enthusiastic to participate longer duration intervention, on the other hand, mothers were uncomfortable to discuss sensitive issues in the group sessions.	Workforce: selection of community health workers: job circular were distrubuted in the villages. Challenge to find candidates due to lack of formal educational level required (secondary school), social norms against women working outside home, disparities in performance in training vs. practice sessions. Selection was based on the performance of community health worker pos training.	Intervention/pilot: One-on-one motivational meetings were organized by the community health workers to encourage attendance. Average sessions attendance was 95% in the combined arm and 80% of the group arm. Expectation of financial incentives led to negative attitudes of family members towards the intervention. Occasional participation of husbands would facilitate their understand on the intervention and increase acceptability.<br><br>Intervention/trial: The mean number of the 18 sessions attended was similar across arms, with 14.2 (SD 4.0) in the group arm, and 15.4 (SD 3.2) in the combined arm. Participants in both intervention arms had a higher prevalence of any children's picture books in the home at intervention endline (control 19%, group 85%, combined 93%), an indication that participants kept the books they received in intervention sessions. The stimulation activities 'played with', 'read books to' and 'sang songs to' werethe stimulation activities that had the highest prevalence differences when comparing the intervention groups to the control.	Not reported	Training/Monitoring- Pilot: The training methods included participatory discussions, demonstrations of the play activities, role playing, and practice conducting sessions with real mothers. Senior psychologist directly observed 25% of the total sessions in both the arm to increase the quality of sessions. Any weaknesses/issues related to optimum session delivery were discussed every week through meeting with full team.<br>Training/Monitoring - Trial: Trainings included didactic sessions, in-class practice and field practice where community health workers given feedback and practiced observing and giving feedback to others. Supervisors observed at least one group session (or three individual home visits) per community health worker was supervised during each 2-week period and filled out session monitoring sheets and provided feedback to community health workers.	Pilot: Intervention curriculum content was adapted and restructured, emphasizing information for the specific age group present, and adding guidelines for interactive session delivery. A story telling approach was included, focusing more on behavioral activation in real life situations, e.g., practicing productive behavior leading to improved physical and mental health.The session guidelines were adapted to include a summary with key points to deliver and the take home messages at the end of each session.	Not reported	Not reported	Not reported	Not reported	Pilot: Behavior change on mothers were identified such as increased antenatal care visits, childcare activities, rest, and nutritious food<br><br>Trial: Children in the group and combined intervention arms received significantly more stimulating activities in the past 3 days from their primary caregiver, had a larger variety of stimulating play materials in the home, and and had improved scores for the observation of caregiver responsiveness and the child's environment scale. Children in the group and combined arms scored higher than the control arm on all domains of the child development and communicative inventory assessments. Maternal mental health, nutrition, WASH and lead outcomes were improved.	Not reported
	Modified Reach Up	Workforce: Possible evidence that health workers with more education were more respected than local women when providing the intervention.	Intervention: The intervention was feasibly integrated into clinic services.<br>Workforce: Program was initially difficult with staff workload and expectations, but this was addressed by engageing workers and supervisors. Caseload of 25 children-caregiver pairs per week at each clinic was feasible.	Intervention: Group sessions were favored for practicality, efficiency, and acceptability, with emphasis placed on health workers developing good relationships with the mothers. However, mothers's attendence declined over time. Many mothers expressed ambivalence about sessions. Compliance by mothers and staff was good. Mothers appeared comfortable and less inhibited in pairs than in individual sessions. Very few mothers refused to enroll in the program and those who did had reasonable attendance.	Workforce: Health workers attended trainings and ran intervention sessions without additional incentives, although workforce and leadership expressed concerns about workload and motivation.<br>Training: Complexity of the curricula was a barrier for intervention implementation. Health staff were overly didactic during parts of the session that involved discussion.	Not reported	Intervention: Families and communities were involved in adapting the intervention: picture books were redrawn to reflect local context; books were printed locally; local, traditional versions of songs and games were included.<br><br>Workforce: All health workers were trained in the intervention clinics to increase coverage and reduce costs.	Not reported	Training, Supervision: Need for training and supervising to be eastblished within and conducted by the Ministry of Health. To be most sustainable, supervision should be provided by the Bangladeshi Ministry of Health; however, it is unclear if they would have the capacity to do so.	Not reported	Program emergence: Situational analyses identified the existing public infrastructure and the government commitment. Interventions were run through clinics using existing staff. Researchers built on long-standing relationships with local partners and in particular the government health sector.<br><br>Intervention: A few local intervention trials demonstrated the effectiveness and feasibility of responsive caregiving interventions on a small scale.	Intent-to-treat analysis showed a significant improvement in children's cognition, language, and motor composite scores and behaviour ratings, No significant effect on growth and no differential effect of treatment by sex, maternal education, or wealth. The intervention improved home stimulation and maternal knowledge of child-rearing.	Not reported
	Psychosocial Stimulation	Intervention: Intervention deemed potentially appropriate for settings where caregivers are more stable, able to interact with children, and able to receive regular home visits. The intervention was not completely appropriate when delivered in slums where households are unstable and lack structure.	Intervention: Fewer home visits could feasibly still have a positive impact on ECD outcomes. While the original intervention plan reduced visit frequency to monthly for the final three months for feasibility, the researchers changed this to fortnightly due to concerns about lack of effect.<br><br>Workforce: Hiring new health workers was not as feasible as using staff members at primary care clinics to deliver the intervention.	Intervention: While there was still considerable numbers of families not coming back for services, moving services from the hospital to community-based clinics increased follow-up attendance of families with severely malnourished children receiving services.	Not reported	Not reported	Intervention: The number of home visits was increased from monthly to fortnightly for the last 3 months of the intervention to ensure an adequate number of visits.	Not reported	Not reported	Not reported	Program emergence, Intervention: More community-based trials in different setttings are needed to inform the development of a feasible, scaled up program that can be delivered in diverse settings.	Any stimulation (with or without food supplementation) had significant benefits on mental development and weight for age z-score.	Not reported
	Modified Jamaican Home Visiting Program	Intervention: A daily trip to the health center to acquire the nutrition supplement was not appropriate for the local context and population. Additionally, it was not appropriate to only treat one child in the households.	Program emergence: Indication that different ranges of attendance would be feasible.<br>Workforce: Future programs should leverage the existing nutrition infrastructure instead of hiring and training external staff.	Not reported	Not reported	Not reported	Intervention: Focus groups with mothers determined practices, knowledges, and attitudes toward child care and development, which helped identify positive and negative caring practices as well as traditional games and songs that were later incorporated into local material. This included the use of traditional games, songs, and low-cost picuture books suitable for local children and mothers with limited literacy.	Not reported	Not reported	Not reported	Program emergence: Scaling would be more feasible if the intervention was incorporated with the existing nutrition center infrastructure and staff.	Evidence that the intervention may lead to sustained ECD benefits over time.<br>Improvement seen in mothers' knowledge of child rearing and responsiveness.<br>Home visits and group meetings did not lead to significant ECD outcomes.	Neither undernourished group saw improvements in weight or height for age. Children receiving the stimulaiton intervention had significant improvements in mental development, but not physical development. The improvement was about one-third a standard score, which may be limited when taken alone but indicates that the intervention may lead to sustained beenfits over time. The treatment did improve mothers knowledge of child rearing. Intervention children were more responsive, cooperative, happier, and vocalized than control chidlren.
