# Supplementary material for: Pathways to scale up early childhood programs: A scoping review of Reach Up and Care for Child Development
Source: PLOS Glob Public Health. 2023 Aug 9;3(8):e0001542. doi: 10.1371/journal.pgph.0001542 (PMC10411826; doi:10.1371/journal.pgph.0001542)
Supplement: Attachment — Submitted filename: Rebutal letter.docx [file pgph.0001542.s013.docx]

Dear Dr. Steve Zimmerman,

Plos Global Public Health Editor

Thank you for the opportunity to resubmit the PGPH-D-22-01126 “Pathways to scale up early childhood programs: A scoping review of Reach Up and Care for Child Development” after carefully addressing the editor’s comment as described below. We hope the manuscript now meets the standards for scoping reviews outlined for Plos Global Public Health.

Editor’s comment:

While PLOS Global Public Health does consider systematic reviews and meta-analyses for publication, we require that these submissions address a clear and defined research question, conduct a systematic and comprehensive literature review; and use clearly reported, reproducible, and systematic methods to identify, select, and critically appraise relevant research. Our author guidelines for systematic reviews are at <https://journals.plos.org/globalpublichealth/s/submission-guidelines#loc-systematic-reviews-and-meta-analyses>. In the case of your submission, we are concerned that the literature review was completed in July 2020. Systematic and scoping reviews should provide an up-to-date overview of the research topic covered. Since the literature search was completed over a year ago, we are concerned that this study does not meet this requirement. As a result of these concerns, we cannot consider the manuscript for publication at PLOS Global Public Health at this time. However, if you are able to address the concerns discussed above, we may be able to consider a revised version of the manuscript. This would however require a substantial amount of work and thus, would be considered a new submission. If you are able to revise the manuscript to respond to the concerns noted above, please refer to the original submission in the covering letter and accompany the submission with a rebuttal letter that responds to the comments made on the original manuscript.

Author’s response:

As requested, we updated the scoping review search as of July 16, 2022. The search update is indicated on page 6, line 111 of the revised manuscript. A total of 266 new records were found; out of which, 91 were duplicates. Thus, 175 records were screened for eligibility criteria, 105 were excluded by reviewing the abstract, and 70 were excluded after a full-text review. Two study protocols were included after reviewing the citation chain of included articles. In total, 10 new records were added to the scoping review: 6 records corresponding to three new programs one in Tanzania (CCD) and two in Bangladesh (RU), and 4 added implementation information of programs already included in the review, including Mozambique (CCD), Brazil (CCD), Colombia (RU), Bangladesh (RU). After the search update, results, figures, tables, and references were updated accordingly and we now hope the manuscript meets the standards for scoping reviews to be considered for peer review.
