# Supplementary material for: Pathways to scale up early childhood programs: A scoping review of Reach Up and Care for Child Development
Source: PLOS Glob Public Health. 2023 Aug 9;3(8):e0001542. doi: 10.1371/journal.pgph.0001542 (PMC10411826; doi:10.1371/journal.pgph.0001542)
Supplement: Attachment — Submitted filename: ResponseLetter-2.12.2023.docx [file pgph.0001542.s014.docx]

To the Editor and reviewers of Plos Global Public Health:

Thank you for taking the time to review our manuscript titled: “Pathways to scale up early childhood programs: A scoping review of Reach Up and Care for Child Development” and provide detailed and constructive feedback. We reviewed the comments with great interest and revised the manuscript accordingly. Attached is a point-by-point response to the reviewer.

Thank you so much for the opportunity to resubmit this revised version. We hope our revised version meets your expectations.

Kind regards,

The authors.

----------------------------------------------------------------------------------------------------------

Journal Requirements:

1. Please review your reference list to ensure that it is complete and correct. If you have cited papers that have been retracted, please include the rationale for doing so in the manuscript text, or remove these references and replace them with relevant current references. Any changes to the reference list should be mentioned in the rebuttal letter that accompanies your revised manuscript. If you need to cite a retracted article, indicate the article’s retracted status in the References list and also include a citation and full reference for the retraction notice.

Response: We reviewed the reference list and confirmed it is complete and correct.

2. Please amend your detailed Financial Disclosure statement. This is published with the article. It must therefore be completed in full sentences and contain the exact wording you wish to be published.

a. State the initials, alongside each funding source, of each author to receive each grant.
b. If any authors received a salary from any of your funders, please state which authors and which funders.

If you did not receive any funding for this study, please simply state: “The authors received no specific funding for this work”.

Response: We reviewed the Financial Disclosure statement and added the information requested.

3. Please ensure that Funding Information and Financial Disclosure Statement are matched.

Response: We confirmed Funding Information and Financial Disclosure statement are matched.

4. We do not publish any copyright or trademark symbols that usually accompany proprietary names, eg  ©, ®, ™  (e.g. next to drug or reagent names). Please remove all instances of trademark/copyright symbols throughout the text, including ™ on page 7.

Response: We removed it from page 7.

5. Fig 2: please (a) provide a direct link to the base layer of the map (i.e., the country or region border shape) and ensure this is also included in the figure legend; and (b) provide a link to the terms of use / license information for the base layer image or shapefile. We cannot publish proprietary or copyrighted maps (e.g. Google Maps, Mapquest) and the terms of use for your map base layer must be compatible with our CC-BY 4.0 license.

Response: Done. We retrieved the base geography layers from Natural Earth (<https://www.naturalearthdata.com/downloads/10m-cultural-vectors/>). A direct link to the base layer of the map was included in the figure legend. The terms of use/license follow Plos Global Public Health requirements and can be found at: <https://www.naturalearthdata.com/about/terms-of-use/>

----------------------------------------------------------------------------------------------------------

Review Comments to the Authors:

Reviewer #1: This study provides a timely and critical summary of the implementation pathways of two widely-used nurturing care interventions. The authors use the findings of this review to provide a clear and necessary checklist to guide program implementers in their decision-making processes. I have included some comments in my review on minor points the authors can address to further improve the readability of their manuscript.

Response: Thank you for the positive feedback. We truly hope that this manuscript will support decision-makers and program implementers in scaling up quality ECD programs.

1. Please review the manuscript to correct minor spelling and grammar mistakes.

Response: We carefully proofread the manuscript for spelling and grammar mistakes.

2. Results (pages 13-25) – When describing the implementation strategy themes, it would be helpful to have specific examples of some themes in the text (e.g., type of training skills used to train implementation agents) instead of putting all of those details in the tables. Right now, there is a lot of key information in tables, and it is difficult to go back and forth between the text and tables to clearly understand the results.

Response: We have included one example for each implementation strategy theme identified in the results section (pages 14-28)

3. Results (pages 13-25) – Likewise, it would also be helpful if the authors provided more information on implementation outcomes including their definition (to ensure that readers and the authors have similar understandings of each term) and how they were assessed across studies.

Response: We have added a new Table 1 providing the definition of implementation outcomes used to assess qualitatively these outcomes across studies (page 8).

4. The authors should consider including Luoto et al’s (2021) recent implementation evaluation paper (published in Frontiers in Public Health) in the review

Response: Thank you for this recommendation. We reviewed the manuscript by Luoto et al manuscript and it did not use the Care for Child Development or Reach Up approach. For this reason, we incorporated this implementation study into our discussion section (page 38).

5. Figure 10 – the details of the attached image are difficult to read, please provide a higher-quality image.

Response: Done.

6. Discussion (page 36, lines 498-507) – The proposed ECD Program Implementation Checklist provides a helpful guide for decision-making on program implementation strategies. The authors do recommend the use of this checklist in conjunction with guidelines such as TIDieR and STaRI to reporting a program’s implementation details; however, these guidelines are limited with respect to reporting of implementation processes, intervention theory of change, fidelity, and program adaptation. Perhaps the authors should consider including the CARE guidelines (Yousafzai et al., 2018, Annals of the New York Academy of Sciences) which include this information and are more specific to nurturing care interventions for promoting early child development.

Response: We appreciate this recommendation. We have added the Consolidated Advice on Reporting ECD implementation research (CARE) guidelines for improving report of ECD interventions in the discussion section (page 40).
