# Supplementary material for: Pathways to scale up early childhood programs: A scoping review of Reach Up and Care for Child Development
Source: PLOS Glob Public Health. 2023 Aug 9;3(8):e0001542. doi: 10.1371/journal.pgph.0001542 (PMC10411826; doi:10.1371/journal.pgph.0001542)
Supplement: Attachment — Submitted filename: Revisions letter-June 27.docx [file pgph.0001542.s015.docx]

Dear Editor and reviewers,

We thank you for your careful feedback. We revised the manuscript accordingly and provide a point-by-point response below:

Reviewer #1: Thank you for addressing the comments raised and making the necessary revisions. This updated manuscript is improved and nearly ready for publication. During the revision process, another (systematic) review on the implementation and evaluation of CCD programs was published (Ahun et al., 2023, Frontiers in Public Health). I suggest that the authors read this new review and revise their manuscript accordingly to build on its findings and avoid unnecessary repetitions across publications.

Response: We reviewed it with great interest. It has been added to the background and discussions sections highlighting how our review and analysis are quite different and adds much more implementation science depth than this review (lines 76-81, 544-552)

Reviewer #2: This was quite a comprehensive and very useful summary of the Reach Up and Care for Child Development program implementation pathways. The main outcome appears to be an ECD implementation checklist with six building blocks, which flows well from the implementation pathways identified in the study. The authors also take care in noting the dearth of implementation details in the literature, and how this gap could affect findings. It seems as if many of the first reviewer’s comments were addressed already, so I have relatively few very minor comments. I think that the paper is fine to publish once the editor is satisfied that the reviewer comments were dealt with.
1. Re-read to correct minor spelling and grammar errors.

Response: The manuscript was reviewed carefully for spelling and grammar by an English native speaker with strong expertise in ECD. All editorial changes have been tracked.

These include but are not limited to:
a. Line 89: “resources constrained settings” should be “resource-constrained settings”

Response: Done (lines 95)

b. Line 90 should be: “… have been no previous attempts…”

Response: Done (lines 93-94)

c. Line 175: “A code structure ……, was” rather than “were”

Response: Done (lines 182)

d. Line 356: should be “…e.g. assess of skills…”

Response: Done (lines 359)

e. In Table 10 on page 31: IS2.5 “delivery sites” rather than “delivery site”. IS3.4 should be “only to” rather than “to only”

Response: Done (page 43-44)

f. Line 473: “befit form” should be “benefit from”.

Response: Done (lines 476)

g. Line 491: scale-up should be hyphenated as it’s a noun.

Response: Done (lines 496)

h. Line 520: fille din should be filled in.

Response: Done (lines 531)

i. Line 563: remove the full stop after Acknowledgments.

Response: Done (lines 574)

j. Line 542: implementors should be implementers as in the rest of the text.

Response: Done (lines 578)

2. Figure 3 is still illegible. Perhaps redo it in landscape and spread it across 2 or 3 pages?

Response: Following the suggestions, we restructured it into a landscape Table. Tables 2a-b.

3. Figures 3 to 9 are text rather than figures. Why is this?

Response: Following the suggestions, we have moved this content to Textbox Tables. Tables 5b-10b.

4. Counting up the n’s in lines 203 to 209 gives me 17 CCD and 13 RU but the text says there should be 18 and 15 respectively. The map in Figure 2 also appears to have 17 and 13. Rewrite the text so it is clear how the total n’s match up to the stated totals in line 203.

Response: We have made the corrections in the text (lines 214, 218) and updated Figure 2.
